# The Andean *Paepalanthus pilosus* complex (Eriocaulaceae): a revision with three new taxa

**DOI:** 10.3897/phytokeys.64.6864

**Published:** 2016-06-13

**Authors:** Nancy Hensold

**Affiliations:** 1 Keller Science Action Center, The Field Museum of Natural History, 1400 S. Lakeshore Drive, Chicago, IL 60605-2496, USA

**Keywords:** Andes, cushion plants, diaspores, leaf anatomy, nectaries, new species, paramo, taxonomy

## Abstract

A herbarium-based revision is provided for *Paepalanthus pilosus* and allies, five commonly confused species of cushion plants native to Andean paramo. These are placed in the recircumscribed Paepalanthus subsect. Cryptanthella Suess. The group includes *Paepalanthus pilosus*, *Paepalanthus dendroides*, and *Paepalanthus lodiculoides*. An additional two species and one variety are newly described: *Paepalanthus caryonauta*, *Paepalanthus huancabambensis*, and Paepalanthus pilosus var. leoniae. The latter two are Peruvian endemics, while *Paepalanthus caryonauta* is known from four countries, and has long been confused with other species. An additional, possibly undescribed taxon is noted from the Serrania de Perijá, Colombia. Five new synonyms and three lectotypes are proposed, and the common misapplication of some names is noted. Within the *Paepalanthus pilosus* complex, species differences were found in timing of peduncle elongation, sex ratio, and leaf, perianth, diaspore and nectary morphology. Ecological differences are suggested by specimen data and a review of ecological literature. Descriptions, photographs and maps are provided for all species, as is a key to the groups of eriocaulaceous cushion plants from Andean South America.

## Introduction

The Eriocaulaceae (Liliopsida: Poales) are a family of 10 genera and about 1200 species, about 800 of which are Neotropical (The Plant List 2013; Giulietti et al. 2012). They usually occur on wet sunny sites with sandy or otherwise acidic soils, and are most diverse in the central highlands of Brazil and the Guiana Shield of Venezuela. In the Andes, diversity is relatively low. About 34 species, including 29 species of *Paepalanthus* Mart., are currently recorded from Andean montane forest and páramo (ca. 1800–4000 m). These occur from Bolivia northwards, with a few extending to the Talamanca range in Costa Rica. Most are endemic to this region. (Giulietti 2015; Hensold and Hammel 2003; Hensold 2008; Hensold 2014; León-Yanez and Hensold 1999; Brako and Hensold 1993; Tissot-Squalli 1997).

The *Paepalanthus pilosus* complex includes seven of the currently recognized Andean species: *Paepalanthus barkleyi* Moldenke, *Paepalanthus dendroides* (Kunth) Kunth, *Paepalanthus dennisii* Moldenke, *Paepalanthus karstenii* Ruhland, *Paepalanthus lodiculoides* Moldenke, *Paepalanthus pilosus* (Kunth) Kunth, and *Paepalanthus schultesii* Moldenke. Members of this complex are characteristic elements of wet peaty sites in climatically humid páramo and subparamo from Costa Rica to Bolivia. All exhibit the cushion plant growth form, or “pulviniform” habit, found among diverse flowering plant families of high-elevation páramo (Luteyn 1999). The similar aspect of the plants, their reduced foliage and capitula, and the variable form of the cushions in response to environment, can make species recognition difficult, and indeed, misidentifications and misconceptions have been common in the taxonomic, floristic and ecological literature.

These species are also easy to confuse with other Andean cushion plant species, including *Eriocaulon microcephalum* Kunth and the three Andean taxa of Paepalanthus subsect. Dichocladus Ruhland (Paepalanthus dichotomus var. glabrescens Moldenke, *Paepalanthus ferreyrae* Moldenke, and *Paepalanthus muscosus* Körn.), but can be distinguished by floral and seed morphology and other microcharacters, as detailed below. Some of the 16 species of Paepalanthus subg. Platycaulon Mart. endemic to the Andes also proliferate from the base and have been described as cushion plants (“*polsterförmig*”; Tissot-Squalli 1997), but these have larger leaves 5–18 cm long arranged in rosettes, and may be more precisely described as “cushion-rosettes” (*cf.* Aubert et al. 2014a).

This study was originally focused on resolving taxonomy of the Peruvian members of the *Paepalanthus pilosus* complex, but later expanded to a herbarium study of all Andean material at hand. While I attempted to describe all material available to me, including that of Colombia and Venezuela, a detailed study of North Andean material was outside the scope of this work. Both *Paepalanthus dendroides* and *Paepalanthus pilosus* exhibit more complex variation over their range than is found in Peru and Ecuador, and the descriptions may not entirely reflect populations of northern South America. Results from more intensive field or molecular studies may add much to our understanding.

## Materials and methods

Specimens were examined from the herbaria F, MICH, and MO (acronyms by Thiers 2015). Images of additional specimens were examined online via JSTOR (2000 onward), as well as the virtual herbaria maintained by COL (www.biovirtual.unal.edu.co/ICN), P (science.mnhn.fr/all/search), NY (sciweb.nybg.org/science2/VirtualHerbarium.asp), and UDBC (herbario.udistrital.edu.co/herbario/). A few additional specimen images were provided by curatorial staff at US, MO and RB. Specimens examined from online images only are cited with barcode number in brackets. Material from USM was studied from handheld digital camera photos, cited with “photo” in brackets. Specimens are listed by country in a north-south sequence, with Venezuela following Colombia, and within country alphabetically by province in boldface, and then by collector and number. In a few cases where practical, geographic groupings within provinces are also used and these are also in boldface.

Specimen localities were interpreted according to the label description and in some cases, published collecting records, and mapped manually using the Google “My Maps” application. Coordinates of the manually placed markers were retrieved as KML data. Final distribution maps were generated using ArcGIS. Estimated coordinates used for mapping are included in the Suppl. material 2.

The key characters used to differentiate Paepalanthus subsect. Dichocladus from Paepalanthus subsect. Cryptanthella in the key were based on examination of the following vouchers. Paepalanthus dichotomus var. glabrescens: Peru. Amazonas: Chachapoyas, *A. Sagástegui 7454* (F), *H. van der Werff 14940 & 16912* (F), *Wurdack 1388* (isotype, F). *Paepalanthus ferreyrae*: Peru. Cajamarca: Cutervo, *J. Mostacero 1594* (F), Peru. Amazonas: Bongará, *J. Wurdack 1081* (F), *I. Sánchez Vega 10020* (F). *Paepalanthus muscosus*: Colombia. Norte de Santander, *Linden 1330* (isotype, F); Venezuela. Táchira, *Maas & Tillett 5282* (F).

Leaf anatomy of the *Paepalanthus pilosus* complex was studied from median hand sections of dried herbarium specimens. Leaves were boiled, stored in 70% alcohol, then rinsed, hand-sectioned in water, and mounted in 50% glycerine. Anatomical vouchers are as follows. *Paepalanthus caryonauta* Hensold: *Barclay 5176* (F), *Boyle 4219* (F), *Fuentes 13574* (F), *Monteagudo 16143* (F), *Valenzuela 8117* (F); *Paepalanthus dendroides*: *Leon 2243 & 2683* (F), *Vásquez 29038* (F); *Paepalanthus huancabambensis* Hensold: *Sagástegui 16799* (F); Paepalanthus pilosus var. pilosus: *Barbour 3427* (F), *Jørgensen 1817* (F), *2209* (F); Paepalanthus pilosus var. leoniae Hensold: *León 1597* (MO). *Paepalanthus lodiculoides* was not sampled due to difficulty sectioning the very small leaves.

Floral measurements were made by ocular micrometer in a stereomicroscope. Except for the Colombian, Venezuelan and Central American material of *Paepalanthus dendroides* and *Paepalanthus pilosus*, flower samples of all available specimens were measured. Photos of dry flowers, seeds and some capitula were taken with a Dino-Lite AM-413T USB Digital Microscope (Figs 2, 5, 7, 9, 10, 12), and if necessary multiple images aligned and stacked in Photoshop. Seeds were photographed after wetting and re-drying to show hygroscopic structures. Figures 4D and 9B were photographed under a stereomicroscope, and leaf cross-sections (Fig. 3) were photographed under compound microscope. Figures 3F and 9B were compiled with Zerene Stacker software.

Conservation status could be assessed with confidence only for taxa of very broad and very narrow distribution. Confident evaluation according to the IUCN (2014) criteria was difficult due to the patchy nature of suitable habitat in the Andes, my lack of fieldwork, and insufficient knowledge regarding rate of habitat loss and other threats. For the taxa not assessed, I offer only notes which may be relevant to the subject, such as a rough estimate of distributional range.

## Morphology

### Habit

**Figure 1.**
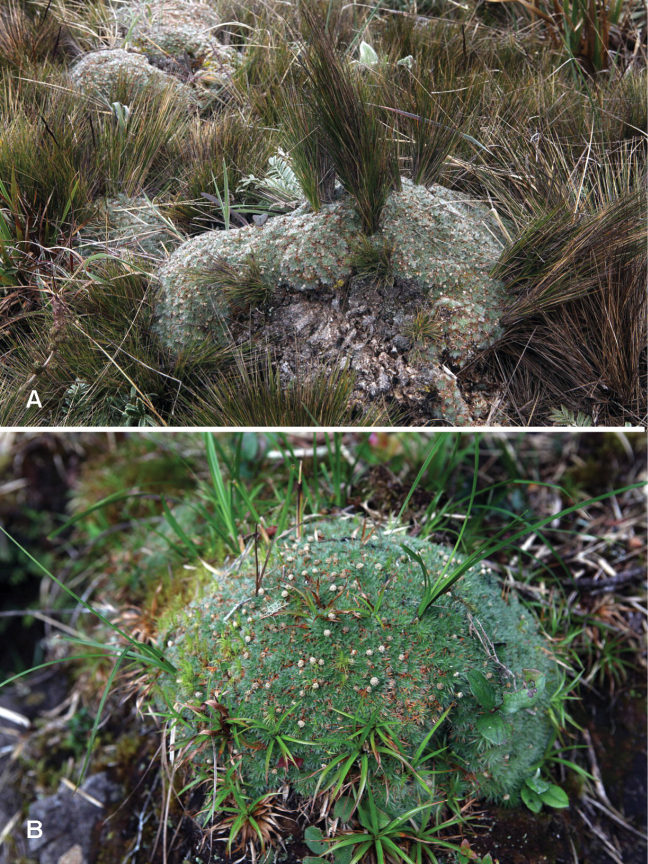
Cushion habit of Paepalanthus pilosus var. pilosus in Venezuela, November 2012. **A** *Paepalanthus pilosus* cushions with bunchgrasses, Páramo Batallón **B** Individual cushion, with graminoids emerging, Páramo Los Conejos. Photos by Serge Aubert, Station Alpine Joseph Fourier, France. Used by permission (www.cushionplants.eu).

All species in the group are perennials with a compact, flat to rounded (pulviniform) cushion habit, formed by short densely branched sympodial stems covered with congested subulate leaves about 1−2 cm long. Inflorescences are solitary and terminal on young shoots, but soon overtopped by erect lateral shoots, and then appear either axillary or borne between two dichotomous branchlets. The cushions may reach up to 1 m in diameter (*Paepalanthus caryonauta*) and 10 cm high (*Paepalanthus pilosus*), with a hemispherical shape (Fig. 1), though they may be much smaller. Aubert et al. (2014b), applying Rauh’s (1939) taxonomy of cushion plant forms, categorized the cushion-forming Eriocaulaceae as the “*Rasenpolster*” type, or “tufted compact cushion,” and presented *Paepalanthus pilosus* (“*Paepalanthus karstenii*”) as an illustration of this growth form. This type of cushion is typical of herbaceous graminoids, in which a central taproot is lacking, and adventitious rootlets issuing from the leaf bases anchor it to the soil. It is perhaps translated more accurately as “turf cushion” (*cf.* Parsons and Gibson 2009). As cushions grow, dead organic matter accumulates in the interior (Fig. 1A, foreground). Later the older growth at the center may rot away, so that the cushion becomes ring-shaped (A. Cleef, pers. comm.). This is illustrated in a photograph by Cleef of Paepalanthus lodiculoides var. floccosus Moldenke in the field (see Moldenke 1976, p. 50).

### Leaf morphology

**Figure 2.**
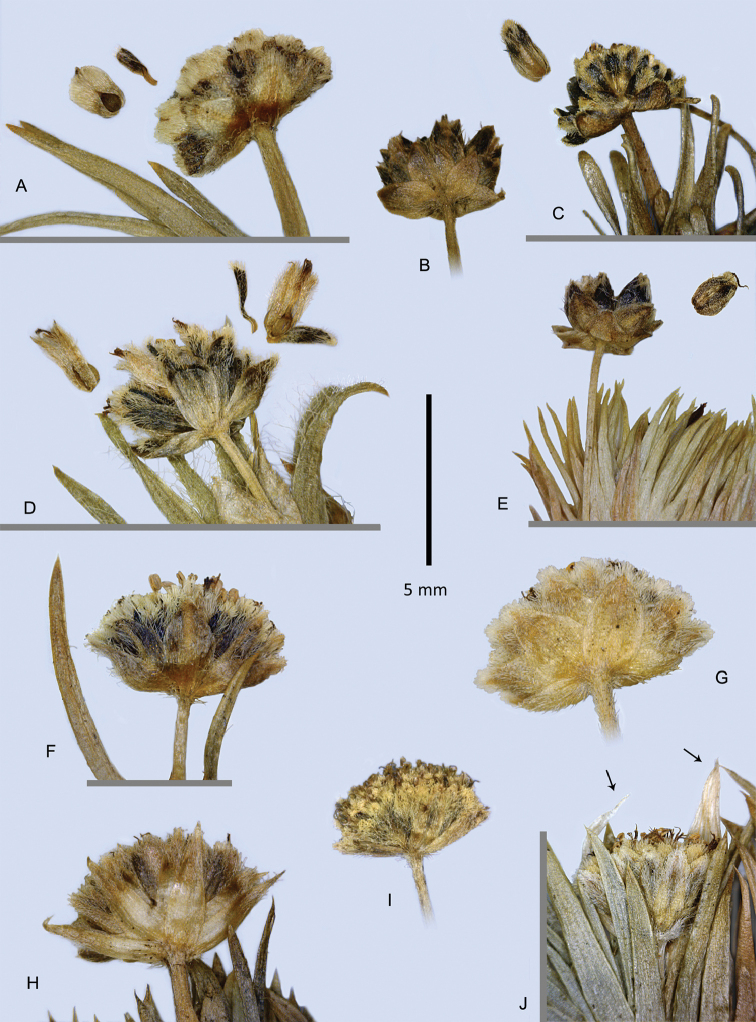
Capitula and leaf tips of *Paepalanthus pilosus* and relatives **A−E** Fruiting capitula with diaspores; sepals detaching (**A, D**) or intact (**C, E**) **A** *Paepalanthus dendroides* **B−C** *Paepalanthus caryonauta* **D** Paepalanthus pilosus var. pilosus **E** *Paepalanthus lodiculoides* **F−J** Paepalanthus pilosus var. pilosus, involucral bract variation. Arrows = tips of the torn peduncle sheath. (**A** *Davidse 28991* **B** *Barclay 5176*, Ecuador **C** *Dudley 11060*, Peru **D** *Cuatrecasas 25574* **E** *Øllgaard 9717* **F** *Cuatrecasas 25882* **G** *Cuatrecasas 5553* **H** *Øllgaard 9557* **I** *Cano 16840* **J** *Jørgensen 2366*.)

Leaf morphology is similar among species, with subtle differences found in color, thickening, and pubescence. *Paepalanthus huancabambensis* is distinguished by a deep blue-green leaf color, while *Paepalanthus dendroides* has leaves distinctly pale green in drying. In other species, leaf color is variable. The upper leaf surface is persistently pubescent in *Paepalanthus huancabambensis* and in *Paepalanthus dendroides* of Huánuco and Cuzco. In *Paepalanthus pilosus* of Peru and Ecuador, appressed pubescence commonly occurs on the upper surface near the apex, while robust scattered cilia extending to the distal margin are typical of plants from the northern part of the range. *Paepalanthus caryonauta* has a rounded leaf apex (Fig. 2C), with the cuspidate tip strongly deflexed if present, while *Paepalanthus pilosus* has leaves acute to sharply aristate (Fig. 2D, F, H, J), and the other species have leaves acute but not aristate (Fig. 2A, E).

### Leaf anatomy

**Figure 3.**
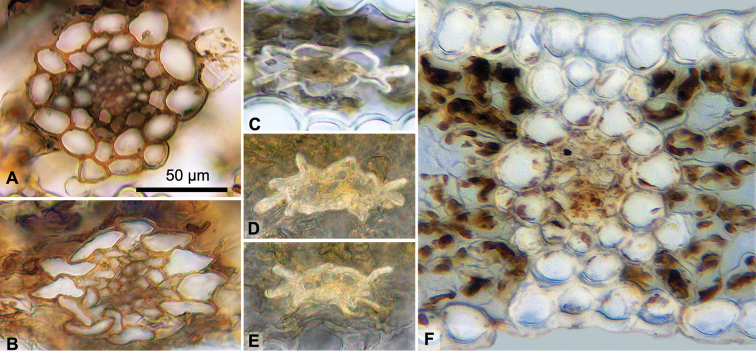
Vascular bundle morphology, adaxial side up. **A** *Paepalanthus caryonauta* **B** Paepalanthus pilosus var. pilosus **C−E** *Paepalanthus dendroides* **F** *Paepalanthus huancabambensis* midvein, with bundle sheath extensions. (**A** *Valenzuela 8117* **B** *Barbour 3427* **C** *León 2683* **D−E** *León 2243*. F: *Sagástegui 16799* **D−F** Mesophyll darkened for contrast.)

The 13 specimens sampled represent all taxa of the *Paepalanthus pilosus* complex except *Paepalanthus lodiculoides*. Their anatomy may be characterized as follows: Epidermal layer one cell thick of large rounded cells, with those of the upper epidermis slightly larger than the lower epidermis; leaf margin rounded, with smaller usually thicker-walled cells. Stomata abaxial only. In *Paepalanthus dendroides*, the epidermal cells larger and mostly thinner-walled than in the other samples, partly collapsed and deformed in section. In other sampled taxa, the outer epidermal wall thickened, and sometimes heavily cutinized on one or both surfaces. Hypodermis absent. Mesophyll of dense to moderately loose short-armed chlorenchyma, with an adaxial palisade layer sometimes discernible. Veins mostly 5–7. Vein buttresses (bundle sheath extensions) commonly absent in median section (Fig. 3A–E), with exceptions. In *Paepalanthus huancabambensis* the three central veins were buttressed adaxially, and also more weakly or by the midvein only to abaxial epidermis (Fig. 3F). In *Paepalanthus caryonauta*, ﻿a weak abaxial midvein buttress was noted in only one specimen, otherwise buttresses absent. All other samples studied lacked vein buttresses in median section, though they likely occur towards leaf base.

Some unusual anatomical features are present which may relate to the daily freeze-thaw cycles of high elevation paramo. First, the mesophyll often detaches cleanly from the epidermis in an intact layer enclosing the vascular bundles. This was conspicuous in the thick leaves of *Paepalanthus caryonauta* and observed to some extent in all sampled taxa except *Paepalanthus huancabambensis*. It can be seen in dry broken leaves, as well as in hydrated sections. Separation of mesophyll from the epidermis is known from petioles and leaves of various frost-resistant species of other families in connection with extracellular ice formation (Levitt 1956). In these species, extracellular ice crystals can form below the epidermis, drawing water from the parenchyma and causing it to shrink. The cells themselves do not freeze and the tissue rehydrates when it thaws, avoiding permanent damage. McCully et al. (2004) speculate that anatomically determined “fault zones” which accommodate subepidermal ice crystals enhance frost resistance in some species.

Unusual deformation of the bundle sheaths is also observed in some species. In *Paepalanthus caryonauta*, *Paepalanthus huancabambensis*, and most *Paepalanthus pilosus*, cells of the bundle sheath (“endodermis” sensu Coan et al. 2002) are regularly rounded or only slightly compressed (Fig. 3A,F). However, in the observed material of *Paepalanthus dendroides* and Paepalanthus pilosus var. leoniae they are irregular in shape, sometimes flattened so completely the inner walls touch, and the cells lateral to the bundles extend outward in characteristic finger-like rays (Fig. 3C–E). This peculiar feature was observed even in young leaves. Partial distortion of the bundle sheath was also observed in Paepalanthus pilosus var. pilosus (Fig. 3B). The semi-aquatic species *Paepalanthus dendroides* tends to have thinner cell walls, which may be expected to deform more easily. However, Paepalanthus pilosus var. leoniae, which occurs at the highest elevation for that species in Peru, in spite of its thicker cell walls, also showed highly distorted bundle morphology, with cells of the bundle sheath and phloem barely discernible. While some distortion of cells is to be expected due to the drying process, the consistent differences observed between taxa growing at similar elevations suggest other factors are involved.

### Timing of peduncle elongation

Paepalanthus pilosus var. pilosus is distinguished, especially in Peru and Ecuador, by the capitula usually subsessile and partly contained within the peduncle sheaths at flowering time (Fig. 2J). However, the peduncles often elongate dramatically in fruit, up to 10 times or more their length at anthesis (Fig. 11D). This delayed timing may serve to protect flowers at anthesis while enhancing fruit dispersal in the extreme páramo habitat. Although peduncle length is known to be variable in this species, the correlation of elongation with fruiting has not been previously noted. In the other species, dwarf peduncles occasionally occur, probably in response to habitat, and slight elongation may occur after flowering, but not to the dramatic degree observed in *Paepalanthus pilosus*.

### Arrangement of staminate and pistillate flowers

Most species have protogynous capitula, with pistillate flowers at the periphery, and staminate flowers in the center. The capitula are about 4-24-flowered and usually strongly determinate, with no central floral primordia found at the start of anthesis. In capitula with relatively more flowers (*Paepalanthus huancabambensis*, some *Paepalanthus pilosus*) the ratio of pistillate to staminate flowers tends to be greater (up to 3:1) whereas in smaller capitula it approaches equality. The related taxon, treated as “*Paepalanthus* sp. A” below, differs by the flowers more numerous (40 or more) and the pistillate and staminate disposed in alternating whorls, with staminate flowers sometimes found in the outer whorl (Fig. 11G). An unusual situation is found in *Paepalanthus lodiculoides*, in which a variable sex ratio may be observed among capitula on the same plant. Wholly staminate capitula are frequent in this species, while bisexual capitula show variable ratios of pistillate to staminate flowers. Wholly pistillate capitula were not observed.

### Nectaries

In *Paepalanthus* and other genera of the Paepalanthoideae, modified style branches may function as nectaries (Stützel 1984, Oriani et al. 2009). These clavate structures have traditionally been termed “stylar appendages,” (e.g., Ruhland 1903), but Stützel (1984), noting their homology to a style branch itself, substituted the term “Drüsen” (glands), and later “gynoecial nectaries” (Stützel and Gansser 1996), while Rosa and Scatena (2007), suggested “nectariferous branches of the style.” Similar secretory structures in the base of the staminate corollas are sometimes termed pistillodes. For simplicity I have used the term “nectary” for these structures, which are similar in both the pistillate and staminate flowers of the species treated here.

In *Paepalanthus lodiculoides* (Fig. 10D) and *Paepalanthus dendroides* (Fig. 7G) nectaries are usually colorless, weak and membranous, curving slightly or collapsing after anthesis. However, in southern Peru, nectaries of *Paepalanthus dendroides* may be light pink or brown. In the staminate flowers of these two species, the nectaries are well included in the corolla, only reaching about halfway to the sinuses of the tube. All other species have rigid dark brownish nectaries, which reach the sinuses of the corolla tube in the staminate flower, and are often well exsert from the pistillate flowers, especially in fruit. In *Paepalanthus pilosus* (Fig. 12B, D, I) and *Paepalanthus huancabambensis* (Fig. 9I) the papillae rimming the top of the nectary are stiff-walled and mostly colorless, only partially collapsing after anthesis and contrast strongly with the dark-pigmented body of the nectary. Stiff-walled apical papillae may also be developed, to a lesser degree, in Peruvian specimens of *Paepalanthus dendroides*. In *Paepalanthus caryonauta* (Fig. 5G) the apical papillae are less clearly differentiated in texture and color, and the whole structure uniformly dark brown.

**Figure 4.**
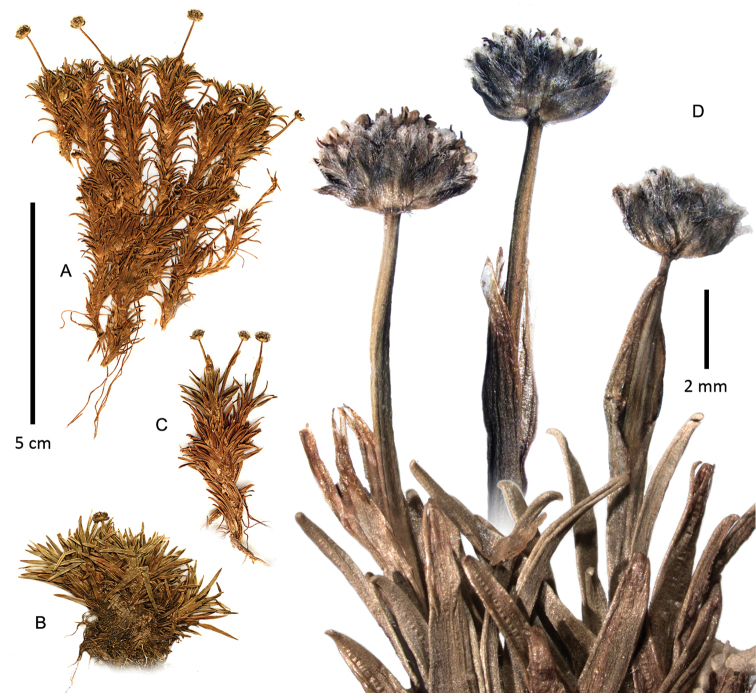
Habit of *Paepalanthus caryonauta* **A** *Boyle 4219* **B** *Fuentes 15374* **C−D** *Valenzuela 8117*.

**Figure 5.**
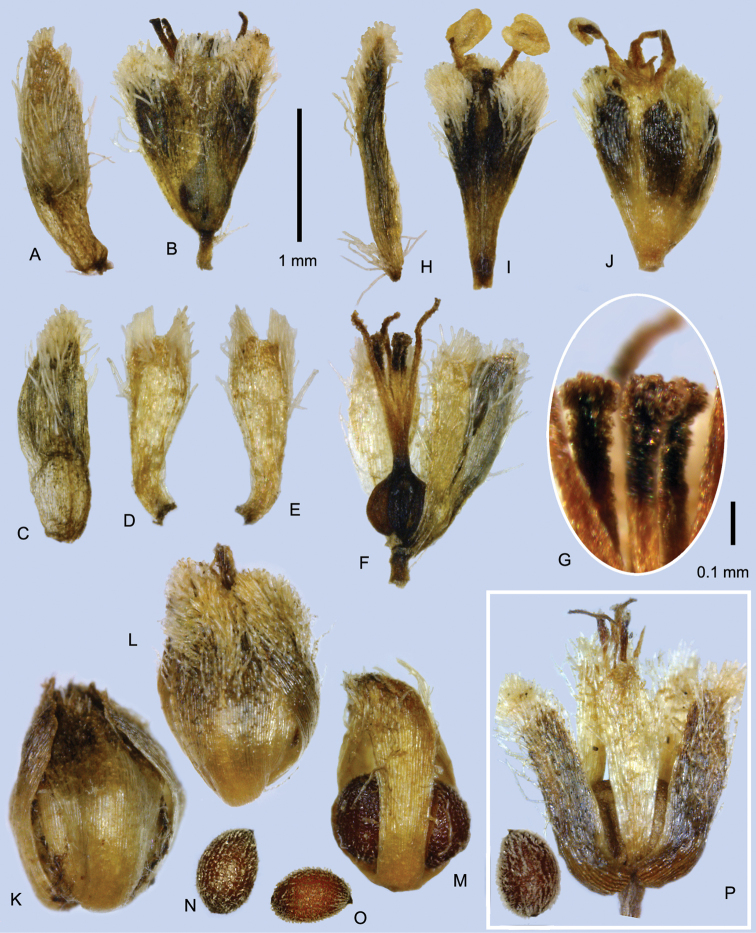
Flowers of *Paepalanthus caryonauta* (**A−O**) and suspected hybrid (**P**). **A−G** Pistillate flowers in anthesis **A** Involucral bract subtending flower **B** Whole flower **C** Sepal (abaxial) **D** Petal, adaxial **E** Petal, abaxial **F** Flower with one petal and two sepals removed **G** Gynoecial nectaries **H−I** Staminate flowers in anthesis **H** Floral bract **I** Flower **J** Staminate flower showing post-anthesis thickening, two anthers fallen **K−L** Mature diaspores **M** Diaspore with sepals removed, with thickened petals **N−O** Seeds **P** Mature flower of probable hybrid (*Roldán 402*), with seed. (**A−I, N** *Valenzuela 8117* **J−K** *Dudley 11060* **L, O** *Boyle 4219* **M** *Dudley 11194*).

### Diaspores

In *Paepalanthus dendroides*, *Paepalanthus huancabambensis*, ﻿and Paepalanthus pilosus var. pilosus the basal half of the fruiting sepals thickens along the midvein at maturity and recurves hygroscopically upon drying, presumably pushing the detached corolla and fruit upward to the capitulum surface (Figs 2A, D; 7E; 12G, H). This is similar to the “elevator mechanism” of dispersal, described by Trovó and Stützel (2011) for *Paepalanthus tortilis* (Bong.) Körn. However, in *Paepalanthus tortilis*, the tips of the sepals recurve sharply, while in the taxa described here, ﻿only the sepal bases thicken and reflex, and the apex remains angled upward. The pilose corolla remains tightly attached to the fruit and is dispersed with it, leaving the sepals behind. In *Paepalanthus dendroides* and to some extent in *Paepalanthus huancabambensis*, the petals are broadly spatulate and densely pilose (Fig. 7), perhaps further facilitating dispersal, while in *Paepalanthus pilosus* the persistent petals are relatively narrow (Fig. 12E, G). However, in *Paepalanthus pilosus* the hygroscopic pseudotrichomes (rod-like epidermal wall remains) of the seed coat are slightly stiffer and more prominent than in any of the other species, while those of *Paepalanthus dendroides* tend to be weak and flaccid (*cf.* Figs 5, 7, 10, 12). Melcher et al. (2004), in a broad survey of paramo taxa, assumed on the basis of morphology that *Paepalanthus pilosus* (“*Paepalanthus karstenii*”) is primarily wind-dispersed and secondarily water-dispersed. They found that diaspores of this species will float for at least three days and suggested that the persistent pilose petals, and perhaps the pseudotrichomes of the seeds, may function to trap air bubbles. SEM photos of the diaspore and seed are provided by Melcher et al. (2004). The diaspores of *Paepalanthus caryonauta* (Fig. 2B–C, Fig. 5K–M), *Paepalanthus lodiculoides* (Fig. 2E, Fig. 10E), and Paepalanthus pilosus var. leoniae (Fig. 12L) are of a different type. In these taxa, both sepals and petals are uniformly thickened in fruit, the broad-based sepals strongly cymbiform-clasping, and the fruit dispersed enveloped by the entire perianth. The fruiting sepals lack any hygroscopic change in shape, and the swollen diaspores detach readily from the capitulum. Sepal thickening is best developed in the Peruvian populations of *Paepalanthus caryonauta*, the thickening extending into the pedicels of the pistillate flowers, which persist as conspicuous “stumps” on the otherwise naked receptacle after flower fall. To compare to Melcher’s results with *Paepalanthus pilosus*, I tested flotation of two diaspores of *Paepalanthus caryonauta* (*Barclay 5136*, *Dudley 11194*), which also floated for three days, probably due to the buoyancy of the thickened perianth tissue. Relative to *Paepalanthus pilosus* and *Paepalanthus dendroides*, however, wind dispersal in this species may be inhibited due to the increased weight and glabrate surface of the diaspore.

The staminate flowers of *Paepalanthus caryonauta* also incidentally thicken with age (Fig. 5J), the corolla developing a thick columnar anthophore, possibly as a pleiotropic effect of the pistillate flower thickening. In the Peruvian plants the staminate flower pedicels are obsolete. In other species, the anthophore is membranous or fleshy but narrowed toward the base, and staminate flowers are normally pedicellate.

### Pathology

Smut fungus infection was observed in both *Paepalanthus dendroides* (*Nuñez 7773*, *Tupayachi 50*) and Paepalanthus pilosus var. pilosus (*Sagástegui 12242*). In these specimens the compact black spore masses swelling the ovary locules simulate mature fruit.

## Ecology and distribution

*Paepalanthus pilosus* and allies occur strictly in wet and very wet paramo and subparamo formations of the Andes, often associated with *Sphagnum*. Most taxa, except for *Paepalanthus dendroides*, occur at about 3100-4000 m on wet but probably not inundated sites. Of these, *Paepalanthus pilosus* and *Paepalanthus caryonauta* are the most similar in habit, both forming dense mats or cushions 30 cm or more in diameter and reported as locally abundant. *Paepalanthus pilosus* is the more commonly collected species, noted for its colonization of disturbed sites, and often cited in ecological studies of paramo as “*Paepalanthus karstenii*.” Further detail is found in the species discussions.

**Figure 6.**
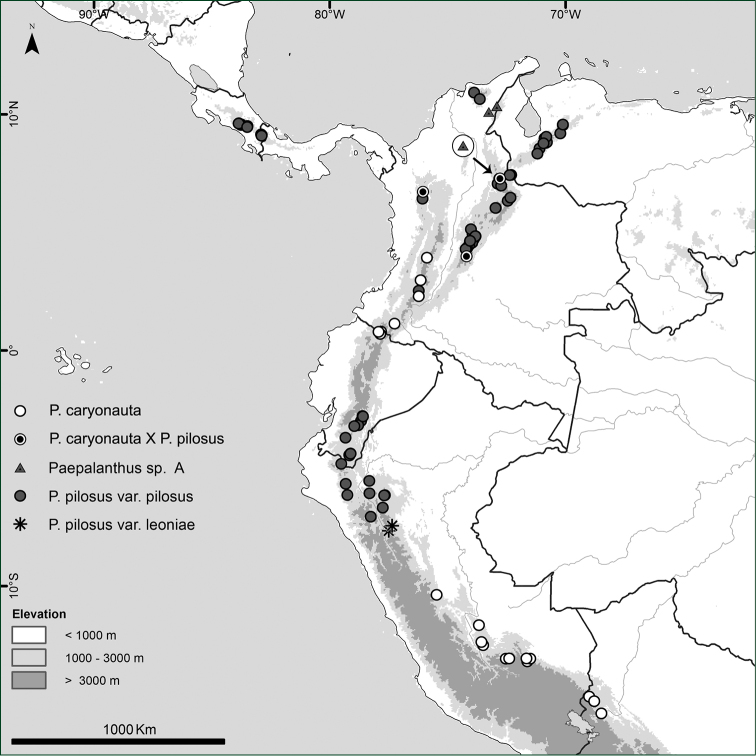
Distribution of *Paepalanthus caryonauta* and suspected hybrids, Paepalanthus pilosus var. pilosus, Paepalanthus pilosus var. leoniae, and *Paepalanthus* “Species A.”

The principal distributions of *Paepalanthus pilosus* and *Paepalanthus lodiculoides*, on one hand, and *Paepalanthus caryonauta* on the other, form an allopatric mosaic with respect to each other (Figs 6, 8). In Colombia, *Paepalanthus caryonauta* is found primarily in the central Cordillera, barely entering northern Ecuador at Páramo Angel, while *Paepalanthus pilosus* is abundant in the eastern Cordillera and the Cordillera de Merida, Venezuela. *Paepalanthus pilosus* reappears in southern Ecuador and northern Peru, from the natural barrier of the Girón-Paute valley (cf. Jørgensen et al. 1995), continuing south through the Amotape-Huancabamba zone (*sensu* Weigend 2002, 2004). *Paepalanthus caryonauta*, in turn, is found at several localities from Central Peru south to La Paz, Bolivia, on the wet eastern slope. *Paepalanthus lodiculoides*, ﻿though less widespread, has a disjunct distributional pattern in South America which parallels that of *Paepalanthus pilosus*.

**Figure 7.**
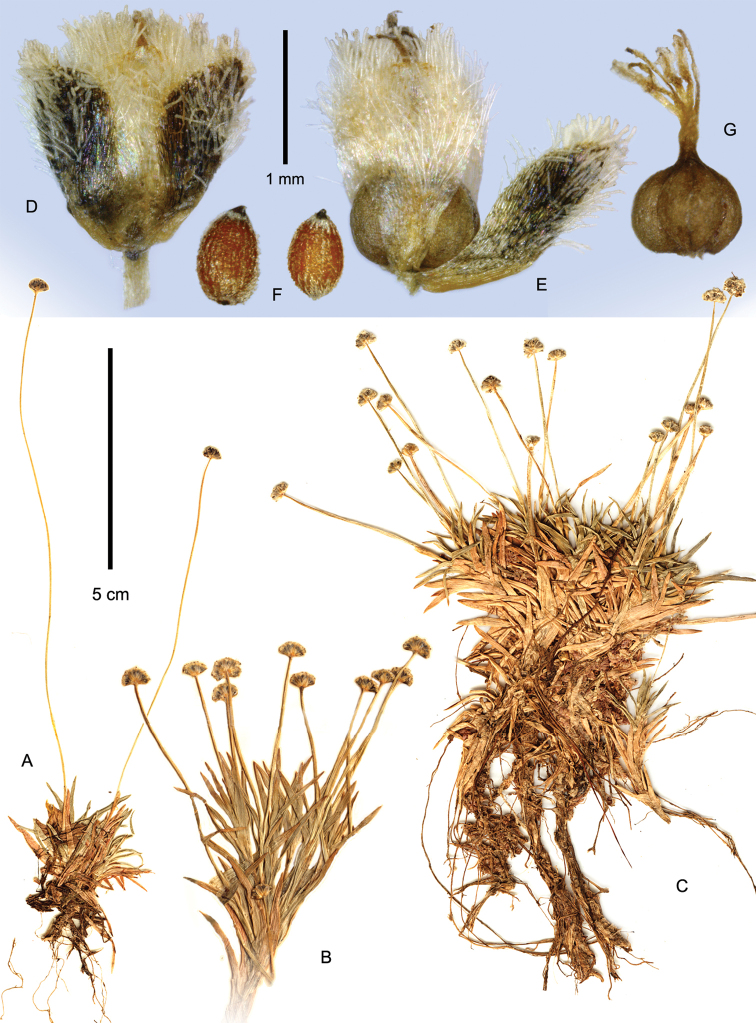
*Paepalanthus dendroides*. **A−C** Habit (**A** *Núñez 7773* **B** *Cuatrecasas 23654* **C** *Davidse 28991*) **D−F** Pistillate flower in fruit (*Pennell 13866*) **D** Whole flower **E** Diaspore with two sepals removed **F** Seeds **G** Gynoecium of young fruit (*Luteyn 10737*).

**Figure 8.**
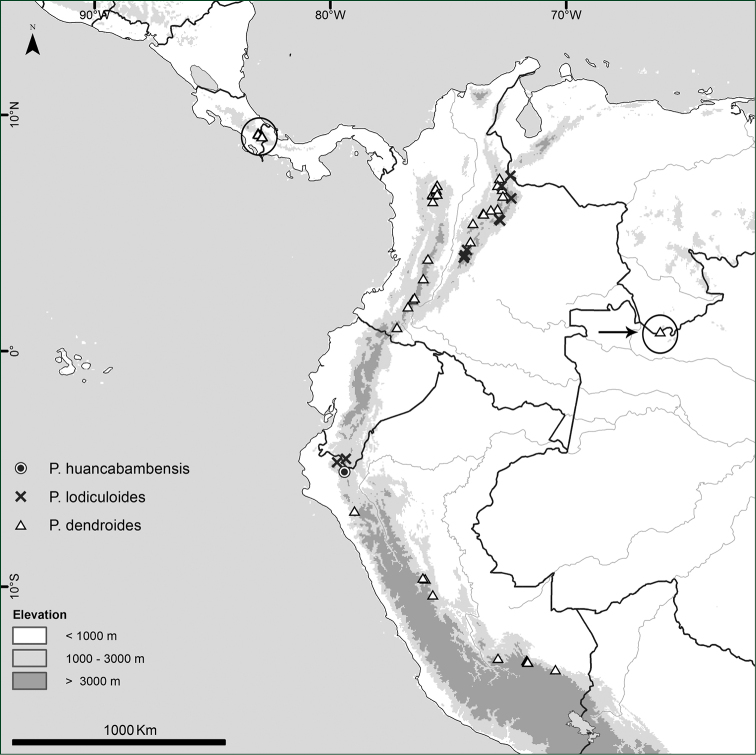
Distribution of *Paepalanthus dendroides*, *Paepalanthus huancabambensis*, and *Paepalanthus lodiculoides*.

Parallel disjunctions may be noted in other pulviniform Andean Eriocaulaceae. The Andean species of Paepalanthus subsect. Dichocladus have a collective distribution similar to that of *Paepalanthus pilosus*, ﻿occurring in the northern part of the eastern Cordillera of Colombia (*Paepalanthus muscosus*), and in the Amotape-Huancabamba Zone (Paepalanthus dichotomus var. glabrescens, *Paepalanthus ferreyrae*). On the other hand, *Eriocaulon microcephalum*, which occurs in the Central Cordillera of Colombia and northern Ecuador, as well as in central Peru has an Andean distribution paralleling that of *Paepalanthus caryonauta*, but also touching the edges of the Amotape-Huancabamba Zone. In Ecuador, it reaches to southern Azuay (specimen data from TROPICOS 2015), but mostly occurs to the west of the Azuay populations of *Paepalanthus pilosus*, on the opposite side of a presumed Pleistocene glacial migration barrier (Jørgensen et al. 1995, Fig. 3; Lozano et al. 2009, Fig. 1). From central Peru it reaches north into San Martín, where it is sympatric with Paepalanthus pilosus var. leoniae.

*Paepalanthus dendroides* has a wide patchy range that overlaps that of both *Paepalanthus caryonauta* and *Paepalanthus pilosus* (Fig. 8), but generally occurs at lower elevations than the other taxa, mostly at 2400-3200 m, but as low as 1900 m in Antioquia, Colombia (*Luteyn 10737*) and up to 3800 m in southern Peru. It is the only species sometimes described as an emergent aquatic. It is also the only species of the group to be collected from the mountains of the Guiana Shield (Pico de Neblina, Venezuela-Brazil border). Together with *Paepalanthus pilosus* it extends to Panama and Costa Rica as well, perhaps reflecting the adaptation of the lightweight comose diaspores of these species for long-distance dispersal.

## Hybridization

A number of suspected hybrids were detected in areas of sympatry. These include a sterile intermediate between *Paepalanthus dendroides* and *Paepalanthus caryonauta* in Pasco, Peru, discussed under *Paepalanthus dendroides*. In addition, material morphologically intermediate between *Paepalanthus caryonauta* and *Paepalanthus pilosus* has been collected in Colombia in areas marginal to the distribution of both species. At the Paramo de Frontino in the western Cordillera, an apparently fertile intermediate plant was found sympatrically with normal *Paepalanthus pilosus*, and a similar intermediate, with abortive flowers, was collected at the south end of the eastern Cordillera. Typical *Paepalanthus caryonauta* is not known from either locality. (See *Paepalanthus caryonauta* discussion.) Finally, as discussed under *Paepalanthus pilosus*, long-term introgression with *Paepalanthus dendroides*, or perhaps another local taxon, is suspected as a factor contributing to the unusually broad, and perhaps bimodal pattern of variation observed in *Paepalanthus pilosus* in disturbed paramos near Bogotá, Colombia, but more study is needed.

## Taxonomic affinities and placement

Ruhland (1903) placed *Paepalanthus pilosus* (including *Paepalanthus dendroides*) and *Paepalanthus karstenii* in *Paepalanthus* series *Leptocephali* Ruhland, an artificial group of reduced mostly annual species with erect stems and terminal fascicles of inflorescences. However, *Paepalanthus pilosus* and allies are perennial cushion plants with inflorescences borne singly, and are clearly out of place here. This misinterpretation may have been due to the fragmentary material available to Ruhland, as well as to Kunth’s mischaracterization of *Paepalanthus dendroides* as having inflorescences in terminal umbellate clusters.

A convenient alternative placement for these plants is available in the overlooked subsection Paepalanthus sect. Paepalanthus subsect. Cryptanthella Suess., described to accommodate *Paepalanthus kupperi* Suess. (syn. of *Paepalanthus pilosus*) from Costa Rica. Suessenguth (1942) compared the new subsection to Paepalanthus subsect. Dichocladus Ruhland, a group characterized by its subdichotomously branched cushion habit. He distinguished Paepalanthus subsect. Cryptanthella by the subterranean branching, with only the erect unbranched rosettes borne above ground. Solitary axillary peduncles were said to arise from near the base of these aerial stems, in comparison to Paepalanthus subsect. Dichocladus, in which inflorescences are borne in the axis between paired dichotomous branchlets (Ruhland 1903). In fact, a similar sympodial branching pattern is found in both groups, with the peduncles terminal on erect leafy shoots at the time of initiation, but overtopped by one or two lateral shoots early in development. The apparent difference in habit is attributable to the tendency in *Paepalanthus pilosus* to form prostrate mats, in comparison to the erect cushions formed by the more rigid stems and leaves in Paepalanthus subsect. Dichocladus. However, the latter group differs sharply from the *Paepalanthus pilosus* complex in a number of other characters (see key below), so Paepalanthus subsect. Cryptanthella is provisionally recognized as a distinct taxon, with a new description and circumscription here provided.

The species of Paepalanthus subsect. Cryptanthella formally treated here are all Andean cushion plants. However, *Paepalanthus pilosus* is very similar to a robust long-pedunculate taxon from páramo in the Serrania de Perijá, Colombia, discussed below under *Paepalanthus* species A. Further affinities of the group are not clear. It was not sampled in recent cladistic studies (Andrade et al. 2010; Trovó et al. 2013), which found a deep division of *Paepalanthus* (ca. 440 spp.) into two major clades not readily distinguished by morphology. Some affinity can be seen with certain long-stemmed species of Paepalanthus sect. Polyactis Ruhland, as to branch architecture, simple style branches and tuberculate floral trichomes. These include *Paepalanthus stuebelianus* Ruhland and *Paepalanthus bongardii* Kunth, both with a more lax, scrambling habit. However Paepalanthus sect. Polyactis also emerged as deeply polyphyletic in the analyses, and the species most similar to Paepalanthus subsect. Cryptanthella were not sampled.

## Taxonomic treatment

Due to the frequent confusion and misidentification of all the Andean cushion plant taxa of Eriocaulaceae, a key distinguishing major groups is provided.

### Key to major groups of Andean cushion plants in the Eriocaulaceae

**Table:** 

1	Roots white, spongy and compressible, easy to tear or cut, septate (with transverse partitions); leaves fenestrate at base (“windowed,” with transverse tissue partitions between the veins); leaf tips acuminose, membranous, pale or discolored; peduncles and outer involucral bracts glabrous; anthers deep black; petals with a black gland on inner apex	***Eriocaulon microcephalum* Kunth**
1'	Roots cream to brown, wiry, with persistent fibrous core, not easily broken by hand nor septate, nor leaves fenestrate; leaf tips thickened, rigid, rounded to aristate; peduncles and/or outer involucral bracts pubescent; anthers cream-colored; petals eglandular	**2**
2	Peduncle sheaths lacking or present, and then the tips ciliate around the whole mouth, sometimes glabrate with age, occasionally splitting with age; pistillate flowers with sepals linear-ligulate, the tips recurving or recoiling in fruit; petals tufted at upper margin only, not pilose on outer surface; seeds smooth and shiny even when wetted, pseudotrichomes absent. Plants of sandy and rocky soils of jalca or montane forest openings, ca. 2000–3000 m elevation	**Paepalanthus subsect. Dichocladus** (Andean taxa)
2'	Peduncle sheaths always present, the tips papery-thin and scarious, often splitting deeply into two or three segments before anthesis, glabrous or minutely tufted at apex, never ciliate around mouth; pistillate flowers with sepals elliptic to spatulate, the tips not recoiling in fruit, and petals prominently pilose on outer and often inner surface; seeds with a whitish covering of matted or distinct pseudotrichomes after wetting. Plants of wet paramo or subparamo often associated with *Sphagnum*, ca. 2000–4000 m	**Paepalanthus subsect. Cryptanthella**

### Key to the species of *Paepalanthus* subsect. *Cryptanthella*

**Table:** 

1	1.7–6.0 mm long, less than 0.4 mm wide in the middle; peduncles glabrous at apex; floral sex ratio very variable, ranging from capitula with flowers all staminate to flowers mostly pistillate	**4. *Paepalanthus lodiculoides***
1'	Leaves (5.5–) 6-22 mm long, 0.6–2 mm wide in the middle; peduncles with a dense collar of hairs at the apex surrounding the base of the capitulum; sex ratio less variable, the capitula with the number of pistillate flowers subequaling to three times more than the staminate in number	**2**
2	Leaf apex narrowly obtuse to rounded, if acute, the tip curved under and not evident from above; leaves glabrous in the distal half or nearly so; sepals of the female flowers thickening uniformly at maturity, enclosing the fruit and dispersed with it	**1. *Paepalanthus caryonauta***
2'	Leaf apex acute to sharp-apiculate, never deflexed; leaves commonly appressed-pilose on upper surface near tip or ciliate, rarely glabrous; sepals of the female flowers thickening only in a narrow strap-like zone in the basal half of the midrib, and separating from fruit at dispersal (except Paepalanthus pilosus var. leoniae, Peru, San Martín)	**3**
3	Peduncles mostly < 7 mm, usually shorter than the sheaths at time of anthesis, often elongating in fruit (rarely up to 50 mm at anthesis, *Cano 16840*, Piura); petals of the female flowers oblanceolate, mostly 2.2–6 times longer than wide; style base (below insertion of style branches) 0.5–1.05 mm long; nectaries dark, rigid	**4**
3'	Peduncles 20–130 mm at anthesis; petals of the female flowers broadly spatulate, ca. 1.6–2.3 times longer than wide; style base less than 0.35 mm long; nectaries various	**5**
4	Fruiting sepals of the female flowers with basal half of midrib thickened, otherwise remaining chartaceous	**5a. Paepalanthus pilosus var pilosus**
4'	Fruiting sepals of the female flowers uniformly thickened throughout and enclosing corolla and fruit at maturity. Plants of San Martín, Peru	**5b. Paepalanthus pilosus var. leoniae**
5	Leaves pale green; peduncle sheaths 11–23 mm, closely appressed, often hidden among the leaves; involucral bracts paler at least along the midvein; nectaries colorless to pinkish or light brown, those of the staminate flowers usually only half-equalling the corolla sinuses (except *Vasquez 29038*, Pasco)	**2. *Paepalanthus dendroides***
5'	Leaves dark blue-green; peduncle sheaths 25-30 mm, lax and open, much exsert from leaf mat; involucral bracts blackish brown throughout; nectaries dark brown, rigid, exsert in fruit, those of the staminate flowers equalling the corolla sinuses	**3. *Paepalanthus huancabambensis***

### Paepalanthus subsect. Cryptanthella

Taxon classificationPlantaePoalesEriocaulaceae

Suess.

Paepalanthus subsect. Cryptanthella Suess., Bot. Jahrb. Syst. 72(2): 293. 1942.

#### Type.

*Paepalanthus kupperi* Suess.

Plants cespitose or pulviniform, forming densely branched clumps with erect terminal shoots ca. 1-4 cm; inflorescences solitary, terminal, but soon overtopped by one or two sympodial branches; peduncle sheaths scarious, swollen at apex, usually splitting into two or three triangular segments; involucral bracts pale greenish to gold or blackish-brown, pilose, not or barely surpassing flowers. Trichomes of the involucral bract and sepal apices subacute to rounded at apex, tuberculate; apical trichome tuft of bracts and sepals relatively short, surpassing perianth tip by less than 0.2 mm. Pistillate flowers peripheral, the outer subtended by the broad inner bracts of the involucre; staminate flowers central or rarely (*Paepalanthus lodiculoides*) the whole capitulum staminate; the inner flowers subtended by linear receptacular bracts, these often with sub-cucullate tips and carinate-clasping bases; pistillate flowers pedicellate; the petals usually long pilose abaxially with tuberculate trichomes disposed in submarginal bands in the upper 1/2 to 2/3 of petal, and also in two dense tufts just inside the upper margin either side of the apex and securing the stigma, the petal tips not involute after anthesis. Gynoecium with stigmas simple; stigmatic nectaries colorless to reddish or dark brown, usually with a distinctly broadened large-papillate upper rim, the papillae in the basal two-thirds or more usually indistinct and scattered. Staminate flower corolla with anthophore usually at least half the total corolla length, membranous or fleshy, corolla tube with three subacute lobes, non-involute after anthesis; staminal filaments prominently fleshy below the corolla lobes and often (not always) adnate to corolla, flat and membranous distally, the tips sometimes turning red-brown with age; anthers whitish, exsert above the lobes, not retracted after anthesis, and often deciduous. Seeds with longitudinal rows of hygroscopic pseudotrichomes.

### 1. Paepalanthus caryonauta

Taxon classificationPlantaePoalesEriocaulaceae

Hensold sp. nov.

urn:lsid:ipni.org:names:77155457-1

Figs 2B–C , 3A , 4 , 5

#### Diagnosis.

Cushion plants with linear glabrescent leaves 1–2 cm long, rounded at the tip, or if cuspidate, the tip strongly deflexed. Peduncles ca. 9–25 mm, solitary, terminal at initiation, sheaths lacerate at apex, eciliate. Pistillate flowers in outer whorl of capitulum, the sepals broad, persistent, together with the petals enclosing the mature fruit at dispersal, nectaries uniformly dark brown, rigid. Staminate flowers central, with sepals fused ¼–¾ of their length.

#### Type.

PERU. Cuzco: Dist. Huayopata, sector San Luis, bosque primario intervenido, 13°04'S, 72°23'W, ca. 3000–3500 m, 24 Nov 2006, *L. Valenzuela et al. 8117* (holotype: F; isotypes: AMAZ n.v., CUZ n.v., HUT n.v., MO, MOL n.v., USM n.v.).

#### Description.

Densely branched cushion plants, the cushions reported to reach one meter in diameter (*Dudley 11194*), and 15 cm high (*Barclay 5176*). Branchlets 1–5 cm, densely and uniformly leafy. *Leaves* linear-subulate, 10–20 mm long including the open basal sheath 3–4 mm long, 1.2–1.7 mm wide at midpoint, ca. 2.5 mm wide at ampliate base; apex narrowly obtuse to rounded, if sharp-cuspidate the tip deflexed downward and not evident in adaxial view; inconspicuously appressed-pilose (rarely ciliate) in juvenile state, glabrate at maturity except for the irregularly ciliate basal sheath; “dark green and glossy” when fresh (*Dudley 11060*), chartaceous to subcoriaceous, the adaxial surface smooth, the abaxial surface often with margins prominently thickened in older leaves and 1–3 veins more or less salient. *Inflorescences* solitary and terminal, with one to four produced in succession along a sympodially branched axis, the lower appearing axillary. *Peduncles* (7–) 9–25 (–35) mm long, ca. 3-costate, obscurely angled, pale, glabrous to obscurely appressed-puberulent, usually with a collar of longer silvery hairs at apex investing base of involucre. *Peduncle sheaths* (9–) 10–17 (–25) mm long, equaling or surpassing the leaf mat by up to 5 mm, the lamina (tip) of the sheath 3–4 mm long, inflated, scarious, somewhat cucullate, almost closed at the apex in bud before emergence of capitulum, tufted-ciliolate at apex when young, often glabrate, frequently splitting into 3 triangular segments with emergence of capitulum. *Capitula* 3–4 mm in diameter. Involucres subequaling flowers at anthesis; *involucral bracts* 2–3-seriate, the outer bracts triangular-ovate to broadly ovate, dull gold, tinged gray or occasionally blackish on shoulders; the inner bracts usually more strongly pigmented except for the paler midvein; bracts bearded apically especially along upper midvein, the upper margin short-ciliate with proximal cilia slightly longer, often early glabrate abaxially. Bract and floral trichomes obtuse to clavate, subhyaline, obscurely ornamented within and very obscurely tuberculate. *Flowers* about 8–14 per capitulum, the pistillate flowers peripheral, the staminate central, equalling to subequalling the pistillate in number; the receptacle sparingly pilose or pilose only toward center. Receptacular bracts equaling to subequaling flowers, broadly linear-subspatulate, ca. 6 times longer than wide, the apex often slightly cucullate and pubescent as the sepals, the base distinctly carinate, clasping. *Pistillate flowers*: Pedicels ca. 0.1–0.35 mm, thick, glabrous, often becoming callose-thickened in fruit (Peru), leaving characteristic ‘stumps’ on the empty receptacle after abscission of flowers. *Sepals* broadly obovate, strongly cymbiform, with apex convex-acute to obtuse (1.35–) 1.5–2.0 mm long × 0.6–1.0 mm wide (width variable within a flower), (0.25–) 0.35–0.4 (–0.5) mm wide at the base; black-mottled on shoulders, the midvein area paler brown; short-ciliate along upper margin and bearded with longer appressed hairs on upper dorsum; membranaceous to chartaceous at anthesis, enlarging slightly and becoming uniformly thickened, and often rigid in fruit, husk-like and non-hygroscopic, enclosing the corolla and fruit and dispersed with it. *Petals* oblanceolate-spatulate, obtuse to often emarginate or truncate-emarginate, (1.2–) 1.35–1.7 (–1.85) mm long × (.35–) 0.4–0.7 (–0.8) mm wide, ca. 2.2–3.2 (–4.9) times longer than wide, cream to brownish-tinged, with scale-like staminode at base, pilose abaxially near margins of distal half with more or less tuberculate trichomes, also densely tufted subapically within in two patches either side of the apex, which enfold the style branch; like the sepals becoming more or less rigid-thickened in fruit, the staminode also thickening and tightly adherent to both petal and ovary base. *Gynoecium* with ovary 0.5 mm at anthesis, ca. 0.85 mm in fruit; the style base 0.3–0.5 mm long; the nectaries with stalks 0.25–0.65 mm long, glandular portion 0.2–0.35 mm long, very dark brown or dark reddish, subclavate, with a ring of brown membranous papillae ca. 2–3 rows thick at the apex, the whole structure maintaining its shape after anthesis; style branches 0.6–1.0 (–1.2) mm long, usually ca. 0.2 mm longer than nectaries though often developing unequally, the stigmas simple, dark red-brown, non-involute after anthesis. Seeds subglobose to ellipsoid, mostly 0.6–065 mm long, 0.4–0.55 mm wide, red-brown, reticulate with short weak pseudotrichomes, sometimes glabrate. *Staminate flowers*: Pedicels 0.15 mm to obsolete. *Sepals* (1.2–) 1.35–1.75 mm long × 0.5–0.85 mm, usually strongly and unequally fused at base for ¼–3/4 their length, obovate-navicular above, the apex obtuse-angled to broadly rounded, the tubular base often rigid and obconic at maturity; color and pubescence as in the pistillate flowers. *Corolla* including anthophore 1.35–1.9 mm; the anthophore 0.65–1.35 mm long, usually ca. 60–70% the corolla length, ca. 0.2–0.35 mm diameter at base, fleshy and columnar at maturity; the corolla tube 0.35–0.75 mm deep, fleshy towards base especially opposite the filaments, the corolla lobes hyaline to brownish, obtuse, 0.15–0.35 mm, not involute after anthesis; intermediate lobes lacking. *Stamen* filaments with the basal half fleshy, terete and adnate to the corolla, abruptly narrowed and loosely adhering to the lobes above, exsert 0.2–0.5 mm beyond the lobes, the exsert portion dark reddish-brown especially at tip; anthers cream-colored, ca. 0.3–0.35 mm long, usually deciduous after anthesis and not present in fruiting capitula. Nectaries similar to those of pistillate flowers, reaching or slightly surpassing the corolla sinuses.

#### Etymology.

The epithet is taken from the Greek *caryonaute* (nom. sing.), the name given to the “nutshell sailors” in Lucian of Samosata’s tale *True Stories*. It refers to the diaspores enclosed by the thick buoyant perianth.

#### Phenology.

In Peru and Bolivia, collected in early anthesis in November and December, and with older inflorescences in all months from February to September. The dry season here is May to August but mitigated at higher elevations by cloud cover (Boyle 2001; Cano et al. 1995). In Colombia and Ecuador, collected on the wet eastern slopes in the slightly drier periods June to September and January; and on the drier western slopes, in the wetter months of March and December (Rangel-Ch. 2000).

#### Distribution.

Colombia (Central Cordillera): Cauca, Nariño. Ecuador: Carchi, probably Sucumbíos. Peru: Cuzco, Junín, Pasco. Bolivia: La Paz. In addition, some atypical specimens or hybrids (see below) are known from the Western and Eastern Cordilleras of Colombia, in Antioquia, Meta, and Norte de Santander. (Fig. 6)

#### Habitat.

In Peru and Bolivia, this species is restricted to a narrow band of wet paramo-like habitat on the high eastern slope of the Andes, while in Ecuador and Colombia it is found in open wet páramo. It is reported from boggy wet bunchgrass meadows (*pajonal*) with *Calamagrostis* Adans. or *Festuca procera* Kunth, in shallow waterlogged soil of ridgetops and rocky slopes, and in cloud forests and páramo degraded by fire. In Ecuador and Colombia also reported from depressions in *Espeletia páramo*. Boyle (2001) describes the species as common in the Cordillera Vilcabamba (Peru: Cuzco/Junín), forming a cushiony matrix together with mat-forming species of *Xyris* Gronov. and Apiaceae between tussocks of *Calamagrostis*. Elevation (2940–) 3100–4000 m.

#### Conservation notes.

This species is known from two disjunct paramo zones, one about 475 km long in the northern Andes and one 950 km long in the central Andes. However, unlike related *Paepalanthus pilosus*, it is not reported from disturbed areas, and rare outlying populations in Colombia show signs of introgression with *Paepalanthus pilosus*. In the event of climatic drying or warming this species would be vulnerable, especially in the southern part of its range where suitable habitat is narrowly restricted to the eastern slope.

#### Misapplied names.

Paepalanthus karstenii f. corei sensu Moldenke (1983) in part, Hensold (2014), non (Moldenke) Moldenke; *Paepalanthus muscosus* sensu R.C. Foster (1958), Moldenke (1979) in part, Balslev (2001) in part, non Körn.; *Paepalanthus pilosus* Brako and Hensold (1993) in part, non (Kunth in H.B.K.) Kunth.

#### Dicussion.

This species is most similar to *Paepalanthus pilosus*, with a similar cushion-forming habit and similar habitat, and is often confused with that species (or “*Paepalanthus karstenii*”). In his later annotations, Moldenke frequently identified this species as Paepalanthus karstenii f. corei (Moldenke) Moldenke. It has also frequently been distributed as *Paepalanthus muscosus* (subsect. *Dichocladus*), which also has rounded leaf tips. The report of *Paepalanthus muscosus* from northern Ecuador by Balslev (2001) is based on vouchers of both *Paepalanthus caryonauta* and *Eriocaulon microcephalum*. The species recently cited as *Paepalanthus muscosus* (Moscol and Cleef 2009) and *Paepalanthus* sp. (Coombes and Ramsay 2001) in vegetation studies of cushion mires in northern Ecuador thus may correspond to *Paepalanthus caryonauta* in part or full, but confirmation is needed.

*Paepalanthus caryonauta* is readily distinguished from typical *Paepalanthus pilosus* and *Paepalanthus dendroides* by the obtuse leaf tips, and by the sepals uniformly thickened and persistent in fruit, to form an ovoid-ellipsoid diaspore. Even in anthesis, the sepals of *Paepalanthus caryonauta* are about twice as broad at the base as those of *Paepalanthus pilosus* and *Paepalanthus dendroides*. *Paepalanthus caryonauta* can also be recognized by eye due to subtle differences in aspect and leaf orientation, with the leaves commonly flatter and more ascending, i.e., less conduplicate and recurved than is commonly seen in *Paepalanthus pilosus*. Boeke, who collected *Paepalanthus pilosus* and an intermediate form of *Paepalanthus caryonauta* at the same locality (see below), noted that in *Paepalanthus pilosus*, the cushions were “easy to separate” and in Paepalanthus aff. caryonauta, “difficult to separate.” In the Cordillera Vilcabamba Dudley reported cushions up to 3 feet in diameter (*Dudley 11194*). However in disturbed roadside páramo at Acjanaco, Young and Cano (1994) comment on the paucity of cushion plants, and do not recognize *Paepalanthus caryonauta* (“*Paepalanthus pilosus*”) as a significant cushion plant species. For other differentiating characters, see Table 1.

**Table 1.** Character comparison of *Paepalanthus caryonauta*, *Paepalanthus dendroides*, and *Paepalanthus pilosus*.

	*Paepalanthus dendroides*	*Paepalanthus pilosus*	*Paepalanthus caryonauta*
Habitat	Terrestrial or emergent aquatic; 1900–3200 m; to 3900 m in Cuzco	Terrestrial, not in standing water; (2900–) 3100–4000 (–4300) m.	
Leaf apex shape	Convex-acute to acute, cuspidate	Acute, cuspidate to short-aristate	Narrowly rounded; if cuspidate, tip sharply deflexed
Leaf pubescence at adaxial apex; at distal margins	Glabrous to persistently hirsutulous; not ciliate	Glabrous (N*) to pilose (S); often prominent long scattered cilia (N)	Early glabrate; not ciliate
Peduncles	20–130 mm	1.5–8 mm (fl); 20–100 mm (fr)	7–35 mm
Capitulum diameter	3–5 mm	3–6 mm (S); 3–8 mm (N)	3–4 mm
♀ Sepal length	1.2–1.9 mm	(1.4–) 2.0–2.7 mm	1.3–2.0 mm
Sepals in fruit	Hygroscopic thickening along basal midline; detaching from fruit at maturity. (Except Paepalanthus pilosus var. leoniae)		Thickened throughout; non-hygroscopic, enclosing fruit
♀ Petals: Length/Width	L/W = 1.6–2.3 Broadly spatulate, densely pilose along upper margin	L/W = (2–)3–6 Oblanceolate, usu. acute, sparingly tufted	L/W = 2.2–3.5 Oblanceolate-spatulate, obtuse; sparingly tufted
Nectaries	Colorless (N) to pale pink-brown (S); weak, partly collapsed in old flowers	Dark red-brown, rigid, erect and exsert in old flowers.	
Nectary position ♂ flower	Only about half-reaching the corolla sinuses.	Reaching the corolla tube sinuses.	
Seeds	Pink to orange-brown; pseudotrichomes weak	Red-brown; pseudotrichomes separating when wet, remaining erect	

* (N) and (S) refer to northern and southern parts of species distribution.

*Paepalanthus caryonauta* has a more uniform morphology throughout its range than its close relatives. However, in Colombia and Ecuador the plants have less thickening in the leaves and flowers, the presence of a short pedicel in the staminate flowers, and peduncles often shorter at flowering time, approaching those of *Paepalanthus pilosus* in length.

#### Hybridization.

*Paepalanthus caryonauta* and *Paepalanthus pilosus* are mostly allopatric, but in Colombia there are points of contact where intermediates occur. Typical *Paepalanthus caryonauta* is common in the Nudo de los Pastos and Central Cordillera of Colombia, from which only one historical collection of *Paepalanthus pilosus* is known, collected ca. 1842. However at the isolated Paramo Frontino in the Western Cordillera, typical *Paepalanthus pilosus* occurs sympatrically with a morphologically intermediate form of *Paepalanthus caryonauta*. These two elements were treated as “*Paepalanthus karstenii*” and “Paepalanthus karstenii var. corei,” respectively, by Rangel-Ch. and Sánchez (2005). In 1976, both elements were collected from *Espeletia* paramo at Llano Grande, with Paepalanthus aff. caryonauta reported from well-drained hillside (*Boeke & McElroy 269*), and *Paepalanthus pilosus* from *Sphagnum* bog (*Boeke & McElroy 265*). Paepalanthus aff. caryonauta was re-collected at the same locality in 1986 (*Roldán 402*), and *Paepalanthus pilosus* in 1989 (*MacDougal & Roldán 4463*, MO). These intermediate plants produce normal seed but have intermediate diaspore morphology (Fig. 5P). The rounded glossy leaves resemble those of *Paepalanthus caryonauta*,﻿ but those of *Boeke & McElroy 269* have long scattered cilia at the upper margin, a trait otherwise only known in *Paepalanthus pilosus*.

In the eastern Cordillera of Colombia, *Paepalanthus pilosus* is abundant, while only two specimens suggesting atypical *Paepalanthus caryonauta* were confirmed, these from opposite ends of the eastern Cordillera, on east-facing slopes. At the south end, an intermediate plant, similar to that of Paramo Frontino, but with flowers mostly abortive, was collected from the eastern slope of Sumapaz National Park (*S. Diaz-Piedrahita 2608*). This location is just south of the southernmost confirmed Colombian collection of *Paepalanthus pilosus*. To the north, *Cuatrecasas 10302* (F), collected at the “extreme east” of Paramo Santurban (Norte de Santander) may represent *Paepalanthus caryonauta* or a hybrid intermediate, differing by the light gold bracts. Sympatric taxa in this area include *Paepalanthus dendroides*, typical *Paepalanthus pilosus*, and the taxon treated below as *Paepalanthus* sp. A.

In southern Peru, typical *Paepalanthus caryonauta* occurs in mixed populations with *Paepalanthus dendroides* at the entrance to Manú National Park (Abra Acjanaco, Cuzco). Both taxa have been abundantly collected and are readily distinguished in the field (A.Cano, pers. comm.) However, in Pasco (Oxapampa), where both species also occur, a sterile intermediate between the two has been collected. (See discussion of *Paepalanthus dendroides*.)

#### Additional specimens examined

**(paratypes). COLOMBIA. Cauca**: Purace – La Plata, 3200 m, 22 Aug 1957, *Barclay 5176* (F,MO); cabeceras Rio Palo, 3700 m, 3 Dec 1944, *Cuatrecasas 19099* (F). **Nariño**: Mpio. Tangua, Páramo de Las Piedras, 3100–3500 m, 9 Jun 2006, *Baca et al. Y-118* (COL [COL000257193]). **Valle del Cauca**: Cabeceras Rio Tulua, 3280–3380 m, 24 Mar 1946, *Cuatrecasas 20278* (F). **ECUADOR. Carchi**: Paramo del Angel, 3700 m, 23 Aug 1957, *Barclay 5136* (MO), 28 Sep 1959, *Barclay 9374* (MO); Tulcán-Maldonado hwy, km 29, 3845 m, 24 Jan 1977, *Boeke 803* (MO); Paramo El Angel, 3500 m, 4 Jan 1973, *Humbles 6086* (MO); Rd Tulcán–Maldonado, km 38, 4000 m, 4 Aug 1976, *Øllgaard & Balslev 8460* (MO). **PERU. Cuzco: Prov. La Convencion**, Cordillera Villcabamba W slopes, 12°36'S, 73°30'W, 3100–3500 m, 14 Jul 1968, *Dudley 11060* (F), 28 km NE of Hda. Luisiana and Rio Apurimac, 12°30'S, 73°30"W, 3400–3600 m, 17 Jul 1968, *Dudley 11194* (F(2),MO,USM); Huayopata, 7 km from Incatambo, S side of Río Lucumayo, 3430 m, 4 Aug 1982, *Peyton & Peyton 914* (MO). **Cuzco: Prov. Paucartambo**, Parque Nac. del Manú, Alturas de Lali, 3750–3850 m, 18 Jul 1990, *Cano 3872* (F, USM n.v.), *Cano 3873a* (USM [photo]); Acjanaco, Cerro Macho Cruz, 3450 m, 30 Aug 1990, *Cano 4024* (F,USM n.v.), 3400–3450 m, 2 Mar 1991, *Cano 4465* (F p.p., USM [photo] p.p., mixed with *Paepalanthus dendroides*); de El Mirador a Cerro Macho Cruz, 3500–3600 m, 21 Jul 1990, *León & Young 2245* (F,USM); Cerro Macho Cruz, 3 Sep 1990, *León & Young 2431* (USM [photo]); Tres Cruces, 4 Apr 1987, *Núñez 7773* (F p.p., USM p.p., mixed with *Paepalanthus dendroides*); Paso de Tres Cruces, Cerro de Cusilluyoc, 3800–3900 m, 3 May 1925, *Pennell 13864* (F). **Junín/Cuzco**: Prov. Satipo/La Convencion, Cordillera Vilcabamba, Río Ene slope, 11°39'36"S, 73°40'02"W, 3350–3400 m, 8 Jun 1997, *Boyle et al. 4219* (F, USM). **Pasco**: Prov. Oxapampa, Distr. Huancabamba, Parque Nac. Yanachaga-Chemillen, Abra Yanachaga, 10°22'49"S, 75°27'42"W, 2940 m, 2 Dec 2007, *Monteagudo et al. 16143* (F,MO n.v.,USM n.v.). **BOLIVIA. La Paz**: Franz Tamayo, Parque Nac. Madidi, entre Queara y Mojos, Calistía, 14°41'26"S, 68°59'44"W, 3600 m, 25 Feb 2008, *Fuentes et al. 12018* (MO); Bautista Saavedra, Apolobamba, sector Codo, 14°53'06"S, 68°46'35"W, 3274 m, 28 Mar 2009, *Fuentes & Huaylla 13574* (F,MO); Cocopunco, 10,000 ft, 24 Mar 1926, *G.H. Tate 382* (NY); Tolapampa, 10,000 ft, 11 Sep 1901, *R.S. Williams 842* (F, NY).

#### Atypical specimens

**(not paratypes). Intermediates with *Paepalanthus pilosus*: COLOMBIA. Antioquia**: Paramo de Frontino, near Llano Grande, 3450 m, 27 Oct 1976, *Boeke 269* (MO), Urrao, Paramo de Frontino, camino que va del Morro al Quince, 3450 m, 11 Sep 1986, *Roldán et al. 402* (MO). **Meta**: Macizo Sumapaz entre Boqueron del Buque and Laguna del Nevado, 3600 m, 7 Jul 1981, *Diaz-Piedrahita 2608* (COL n.v.,MO). **Norte de Santander**: Paramo de Santurban, 3300-3500 m, 27 Jul 1940, *Cuatrecasas & Garcia-Barriga 10302* (F, see discussion). **Intermediate with *Paepalanthus dendroides*: PERU. Pasco**: Dist. Huancabamba. Sector Santa Barbara, Parque Nacional Yanachaga-Chemillén, 10°12'S, 75°22'W, 3200–3250 m, 27 Jan 2005, *Monteagudo et al. 7938* (F,MO n.v.).

### 2. Paepalanthus dendroides

Taxon classificationPlantaePoalesEriocaulaceae

(Kunth in H.B.K.) Kunth

Figs 2A , 3C–E , 7

Paepalanthus dendroides (Kunth in H.B.K.) Kunth, Enum. Pl. 3: 507. 1841.
Eriocaulon dendroides Kunth in H.B.K., Nov. Gen. Sp. (quarto ed.) 1: 251, t. 59, fig. 2. 1815 [1816].
Eriocaulon dendroides Type: Colombia. Cundinamarca: “Crescit in frigidis montanae planitiei Bogotensis inter Suba et Suacha, alt. 1340 hex.,” Jul 1801, *Bonpland & Humboldt s.n.* (lectotype, here designated: B [B 10 0243900]; syntypes: B [B-W 2366], HAL [HAL 0109752], P [P01762723]).
Paepalanthus barkleyi Moldenke, Phytologia 3: 114. 1949. **Syn. nov.** Type: Colombia. Antioquia: 1 km N of Santa Rosa de Osos, 2600 m, 25 Sep 1948, *S. Posada S.*, *M. Torregrosa & F. Barkley 18A100* (holotype: NY; isotypes: COL [COL000006903], CORD [CORD00002162], LIL [LIL000143], US [US00088317]).
Paepalanthus karstenii var. corei Moldenke, Phytologia 29: 386. 1975. **Syn. nov.** Type: Colombia. Cauca: Above Purace, 11,000 ft, 19 Feb 1944, *E. L. Core 272a* (holotype: NY; isotypes: W n.v., WVA n.v.)
Paepalanthus karstenii f. corei (Moldenke) Moldenke. Phytologia 45: 296. 1980. **Syn. nov.** Type: Based on Paepalanthus karstenii var. corei Moldenke.

#### Type.

Based on *Eriocaulon dendroides* Kunth in H.B.K.

#### Description.

Plants terrestrial or partly submerged, forming densely leafy cushions or mats reported up to 30 cm in diameter (*Fassett 25929*), the erect branchlets ca. 1–5 cm. *Leaves* linear-subulate, 9–22 mm long × 0.85–1.6 mm wide, tip cuspidate-acute, persistently pilose to hirsutulous adaxially with white roughened hairs, sometimes nearly glabrous (Peru, Cajamarca and Pasco), mostly eciliate, consistently paler green than *Paepalanthus caryonauta* or *Paepalanthus pilosus*, with veins slightly salient below. *Peduncles* 20–130 mm long, pale, rigidulous (Peru) or often soft and compressible and then noticeably constricted at apex when dry, glabrous except for an apical collar of sericeous appressed hairs; peduncle sheaths 13–23 mm long, tightly enclosing the peduncle, equaling or scarcely exsert from leaf mat, tufted at apex otherwise mostly glabrous. *Capitula* 3.5–5 mm wide. Involucral bracts about equaling flowers, ovate, similar to *Paepalanthus caryonauta* but the outer bracts more hyaline, paler, sometimes uniformly gold and glabrous except at margins. Trichomes of bract and sepal apices obtuse to clavate, strongly tuberculate. Flowers ca. 12–17 per capitulum, pistillate peripheral, the staminate equaling to subequaling the pistillate in number. *Pistillate flowers*: Pedicels ca. 0.25–0.45 mm long, fine, not thickened at maturity. *Sepals* obovate-spatulate, 1.15–1.85 mm long × 0.65–0.85 mm wide, 0.15–0.25 mm wide at base, blackish-brown, short-ciliate along upper margin and bearded with longer appressed hairs on upper dorsum, the basal half of the midrib hygroscopically thickened, and spadiceous-brown in fruit, the broad distal half of the sepal remaining chartaceous, suberect, detaching from diaspore upon dispersal. *Petals* broadly spatulate, 1.15–1.75 mm long, 0.55–1.0 mm wide, ca. 1.6–2.3 times longer than wide, cream-colored and densely long-pilose with tuberculate trichomes on the abaxial surface flanking the midvein, not thickening, dispersed with fruit. *Gynoecium* with style base 0.15–0.25 mm long, the nectaries 0.55–0.7 (–0.85) mm long, the glandular portion colorless to pale pink to red- or yellow-brown, penicillate, slightly curved after anthesis, the apical ring of papillae colorless (white), thin- to thick-walled; styles 0.75–0.9 mm, mostly thinner and less pigmented than in *Paepalanthus pilosus* or *Paepalanthus caryonauta*. Seeds 0.6–0.75 mm long, orange-brown, the pseudotrichomes weak, erect upon wetting but collapsing soon after (few seeds observed). *Staminate flowers*: Pedicels (0.35–) 0.4–0.6 mm long, fine, membranous, nearly glabrous. *Sepals* 1.1–1.9 mm long × 0.35–0.5 mm wide, narrowed to base and very shallowly fused or if fused up to half the sepal length, not thickening, the calyx base not obconic at maturity, color and pubescence as in pistillate flowers. *Corolla* (1.0–) 1.4–1.7 (–1.95) mm long; the anthophore alone (0.35–) 0.55–0.95 mm long, comprising ca. 40–60% of the corolla length, membranous and ca. 0.1–0.2 mm in diameter at base, 0.3 mm at apex, the lobed tube (0.55–) 0.65–0.9 (–1.0) mm long. Filaments often unpigmented, slightly less exsert than in *Paepalanthus caryonauta*, the base of the anther rarely exsert more than 0.3 mm beyond lobe tips. Nectaries well-included within tube, usually only half-equaling the sinuses.

#### Phenology.

In Central America, flowering from March to April, in the dry season, and from August to September, the wet season, punctuated by a short dry period or *veranillo* (Grayum et al. 2004). In Antioquia, Colombia, collected mostly in rainy season from April to September. Elsewhere in Colombia, from July to February. In Peru, collected in anthesis from January to May (Cuzco, Pasco; wet season), and in August (Cajamarca, Huanuco; dry season); in post-anthesis from March to November.

#### Distribution.

Costa Rica (Cerro de Talamanca): Limón, Puntarenas. Panama: Bocas del Toro. Colombia (Central and Eastern Cordilleras): Antioquia, Bogotá D.C., Boyacá, Cauca, Cundinamarca, Nariño, Santander, Norte de Santander. Peru: Cajamarca, Cuzco, Huánuco, Pasco, Puno. Brazil (Pico da Neblina): Amazonas. (Fig. 8).

#### Habitat and ecology.

A terrestrial or partly submerged aquatic, in open marshy subparamo, low paramo or cloud forest margins, in bogs, wet meadows, seeps, commonly associated with *Sphagnum* or tussock grasses, sometimes shrubs or *Blechnum*. Elevation mostly 2300–3200 m, but as low as 1900 m in Antioquia, and up to 3800 m in Cuzco (Abra Acjanaco), Peru. In the high elevation Cuzco plants seed production does not appear abundant, abortive flowers are common, and smut fungus infection is observed.

#### Conservation status.

The conservation status of this widespread species is presumed to be of Least Concern (IUCN 2014). However it may have recently disappeared from the disturbed paramos near Bogotá, where it hasn’t been collected since 1917. Its lower elevation of occurrence and semi-aquatic habit may make it more vulnerable than its relatives to habitat loss due to disturbance.

#### Taxonomic history.

*Paepalanthus dendroides* was initially described by Kunth and later Körnicke (1863) as having unbranched stems with leaves clustered toward the apex and peduncles “umbellate” or in terminal fascicles, a misinterpretation likely due to the small size of the specimens. In addition, Kunth described both *Paepalanthus pilosus* and *Paepalanthus dendroides* as having bifid stigmas; his sketch of the gynoecium of *Paepalanthus dendroides* is mounted on the lectotype sheet. The published plate shows only 3 bifid stigmas, while the sketch shows three presumed filiform “appendages,” alternating with three thick shallowly bifid “stigmas.” Körnicke (1863) later corrected the description of *Paepalanthus pilosus* to “stigmas simple” but accepted without comment the bifid stigmas of *Paepalanthus dendroides*. I have only seen a scan of the type, which clearly corresponds to *Paepalanthus dendroides* as here treated, and can only assume Kunth either misinterpreted the gynoecial structure as he had done for *Paepalanthus pilosus*, or examined an abnormal flower. There are no species in this alliance or among any Andean *Paepalanthus* of similar habit, which have normally bifid stigmas.

*Paepalanthus dendroides* was placed in synonymy of *Paepalanthus pilosus* by Ruhland (1903), but the specialist Harold Moldenke (fl. 1930's–1980's) generally distinguished the two species in his annotation work, misapplying the name *Paepalanthus pilosus* to *Paepalanthus dendroides* and to lax long-pedunculate specimens of *Paepalanthus pilosus*, while using the name *Paepalanthus karstenii* for most material of *Paepalanthus pilosus*. (See detailed discussion under *Paepalanthus pilosus*.) The name *Paepalanthus dendroides* was removed from synonymy and its identity clarified by Hensold and Hammel (2003), but is still commonly used for long-pedunculate plants of *Paepalanthus pilosus* (cf. Madriñán and Zapata 2001).

#### Misapplied name.

*Paepalanthus pilosus* sensu Ruhland (1903) in part, Moldenke (1953, 1975c, 1979, 1983) in part, Brako and Hensold (1993) in part, Huft (1994), Santa Cruz (2011), non (Kunth in H.B.K.) Kunth.

#### Discussion.

*Paepalanthus dendroides* is a variable species across its range, but may be distinguished from its close relatives by the character syndrome in Table 1. The broadly spatulate and densely pilose petals which enfold the diaspore, together with reduced pigmentation of the gynoecia, filaments and seeds, are characteristic. The subaquatic habit and the lower elevation range are distinctive as well. It tends to have a less congested habit and softer leaves than its relatives, but also may form compact, stiff-leaved cushions with dwarf peduncles on some sites, as in the type of Paepalanthus karstenii var. corei, and then floral characters may be important for identification. It should be noted that key characters useful for Costa Rican material (Hensold and Hammel 2003) do not consistently apply in South America, including the pale-striped sepal midlines, the constricted peduncle apex, and the dorsally glabrous involucral bracts. These characters are variable in Colombia and rare in Peru.

Of the Peruvian collections, those from Huánuco, Cuzco, and Puno share a similar morphology, with leaves relatively narrow and conspicuously pilose above, the outer involucral bracts dusky gray, and the nectaries somewhat pigmented. Leaf pubescence easily distinguishes these populations from sympatric *Paepalanthus caryonauta*. In comparison, specimens from Cajamarca and Pasco have broader, glabrous leaves. The Cajamarca collections were examined only from photos, but the plants closely match Colombian material of *Paepalanthus dendroides* and are from a similar habitat and elevation. The Pasco collection (*Vasquez 29038*) has floral characters somewhat intermediate with *Paepalanthus pilosus* or *Paepalanthus caryonauta*, as follows: petals 2.0–2.3(–3.0) times longer than wide; style base 0.4–0.5 mm; nectaries darker, 0.85–0.95 mm long; styles to 1.0 mm long; nectaries of male flowers almost reaching the mouth of the corolla.

#### Evidence of hybridization.

The Pasco, Peru, specimen (*Vásquez 29038*) was collected very near a plant fully intermediate between it and *Paepalanthus caryonauta*, with abortive locules and stigmas (*Monteagudo 7938*; full specimen citation under *Paepalanthus caryonauta*). Typical *Paepalanthus caryonauta* (*Monteagudo et al. 16143*) is recorded 20 km to the east.

In Colombia, introgression between *Paepalanthus dendroides* and *Paepalanthus pilosus* is suspected in the disturbed paramos east of Bogotá. Although *Paepalanthus dendroides* was originally described from near Bogotá, I have only seen one other typical individual from this vicinity (*Pennell 1997*), collected at Quebrada Chapinero in 1917, the same locality where the type of *Paepalanthus schultesii* (=*Paepalanthus pilosus*) was collected in 1941. *Paepalanthus pilosus* is presently abundant near Bogotá, but seems particularly variable and with an unusual tendency to long peduncles, lax habit, and variably shaped bracts, suggesting intermediacy with *Paepalanthus dendroides* (see *Paepalanthus pilosus* discussion). Typical *Paepalanthus dendroides* is currently found north of Bogotá, where it is mostly recorded from elevations 500–900 m lower than nearby collections of *Paepalanthus pilosus*. In Panamá, however, typical *Paepalanthus dendroides* and *Paepalanthus pilosus* are sympatric at the Cerro Fabrega massif, without apparent intermediates.

#### Selected specimens examined

(of 56 total)**. COSTA RICA. Limón**: Cerro Kamuk, 9°14'30"-15'30"N, 83°03'30"-04'30"W, 2900–3100 m, 23–26 Mar 1984, *Davidse et al. 25928* (F,MO); Atlantic slope, between Rio Terbi and Rio Siní, 9°00'-9°12'N, 82°58'-82°59'W, 2400–2750 m, 13 Sep 1984, *Davidse et al. 28991* (F,MO). **Limón/Puntarenas**: Cerro Kasir, 9°12'N, 83°03'30"W, 2950 m, 22 Mar 1984, *Davidse & Herrera 29339* (F,MO). **PANAMA. Bocas del Toro**: [Fabrega Massif], S of Cerro Itamut, 9°05'40"N, 82°53'06"W, 3200 m, 17 Mar 2006, *Monro & Knapp 5369* (MO). **COLOMBIA. Antioquia**: Mpio. Belmira, Finca El Paramo, 3000–3130 m, 29 Jan 1995, *Fonnegra et al. 5408* (F); N of Las Ventanas, 07 02'N, 75 30'W, 1730–1920 m, 26 May 1984, *Luteyn et al. 10737* (F); Yarumal, Llanos de Cuivá, 06°50'N, 75°30'W, 2700 m, 27 Jul 1986, *Roldán 252* (MO); Bello, Vereda San Felix, 06°21'N, 75°39'W, 3050 m, 7 May 1988, *Zarucchi & Echeverri 6332* (MO); **Bogotá, D.C.**: Chapinero, 2700–2800 m, 18–23 Sep 1917, *Pennell 1997* (F); **Boyacá**: Belen, Huina, 7 May 1959, *Barclay 7629* (MO); 14 km NW of Arcabuco, 2440 m, 20 Aug 1944, *Fassett 25629* (MO), Mpio. Chiscas, sector Duartes Arriba, 2850 m, 8 Jul 2003, *Galindo-T. et al. 1303* (COL [COL000057819]); **Cauca**: Valencia, 3090 m, 24 Sep 1958, *H. G. Barclay 5730* (MO); Cordillera Central, E slope [probably valley of Rio San José, Moscoso], 2980–3000 m, 2 Feb 1947, *Cuatrecasas 23654* (F); Paramo de Paletará, 3000 m, 18 Nov 1968, *Espinal & Ramos 3310* (MO); **Cundinamarca**: Pántanos de Fúquene, 2600 m, Mar 1930, *Pérez-Arbeláez 66* (COL [COL 000223800]); **Nariño**: Putumayo, Paramo de Santa Lucia, 2900–3100 m, 9 Jan 1941, *Cuatrecasas 11866* (F); **Santander**: N of Cerrito, 3200 m, 12 Oct 1944, *Fassett 25929* (MO); Las Vegas, 2600–3000 m, 21– 23 Dec 1926, *Killip & Smith 16064* (F); 11 km NE of Berlin, 3330 m, 18 Jul 1979, *Stuessy & Funk 5612* (MICH); **Santander/Norte de Santander**: Paramo de la Laguna, between Pamplona and Bucaramanga, 2900 m, 26 Feb 1939, *A. H. G. Alston 7333* (F). **BRAZIL. Amazonas**: Pico da Neblina, 2700 m, 21 Aug 1985, *C. Farney & Pessoal do 1° B.F.E. 901* (RB) **PERU. Cajamarca**: Prov. Santa Cruz, Distr. de Pulán, El Progreso, 2700 m, 5 Aug 2006, *L. Santa Cruz 653* (USM [photo]). **Cuzco**: Prov. Paucartambo, Parque Nac. del Manú, Acjanaco, 3400–3500 m, 3 May 1990, *Cano 3361* (F), 2 Mar 1991, *Cano 4437* (F), 3500–3600 m, 21 Jul 1990, *León 2243* (F); Trocha Acjanaco - Macho Cruz, 3350 m, 2 Mar 1991, *León & Huapaya 2683* (F,USM[photo]); Paso de Tres Cruces, Cerro de Cusilluyoc, 3800–3900 m, 9 May 1925, *Pennell 13866* (F); Prov. La Convención, Distr. Santa Ana, Pavayoc, 2600 m, 14 Mar 1953, *Woytkowski 567* (USM [photo]). **Huánuco**: [probably “Saxiapata, in the montana of Pillao and Chacahuasi, ” Aug–Sept 1787], *Ruiz & Pavón s.n*. (MO 1612102); Villcabamba, Hacienda on Rio Chinchao, 6,000 ft, 17–26 July 1923, *Macbride 5182* (F). **Pasco**: Prov. Oxapampa, Dist. Huancabamba, Santa Barbara, 10°20'35"S, 75°39'00"W, 3400–3500 m, 25 Jan 2004, *Vásquez et al. 29038* (F,MO,USM). **Puno**: [Prov. Carabaya, San Gaban], “in summis Cordiller. jugis pr. San Govan,” Jul 1854, *Lechler* in *Pl. peruv., ed. Hohenacker 2206* (P [P01762726]).

### 3. Paepalanthus huancabambensis

Taxon classificationPlantaePoalesEriocaulaceae

Hensold sp. nov.

urn:lsid:ipni.org:names:77155458-1

Figures 3F , 9

#### Diagnosis.

Plants forming loose cushions or mats; leaves blue-green, pilose above, older leaves strongly costate below; peduncle sheaths 25–30 mm, very lax, and strongly exsert from the leaf mat, the involucral bracts dark brown, the heads with over 20 flowers; pistillate flower petals spatulate, pilose; nectaries dark red-brown and rigidulous, those of the male flowers equaling the corolla sinuses.

#### Type.

PERU. Piura: Huancabamba, Jalca de Chiguelas, 5°8.2'S, 79°23.6'W, 3082 m, 19 Oct 2001, *A. Sagástegui et al. 16799* (holotype: F; isotype: HAO).

#### Description.

Plants short-caulescent, with erect actively growing shoots to 4 cm, densely leafy, branching to form rounded mats. *Leaves* subulate, acute, 13–22 mm long × 1–2 mm wide at midpoint, tip cuspidate to apiculate, densely pilose to villous above with appressed to spreading white tuberculate hairs, upper margin eciliate, lamina dark blue-green, mature leaves prominently 3–5-costate below. Inflorescences solitary and terminal, soon overtopped by one or two erect lateral shoots which may flower in rapid succession, so that peduncles superficially appear fascicled. *Peduncles* 6.0–11.5 cm long at anthesis, perhaps continuing to elongate into fruiting, with peduncle of previous season up to 15 cm observed on same plant, ca. 3-costate, densely subappressed-villous especially above, with a dense sericeous collar of trichomes subtending involucre. *Peduncle sheaths* 25–30 mm, much surpassing the leaves, and strongly surpassing the leaf mat, scarious, very lax, nearly glabrous except for the tufted apex, the lamina cucullate, enclosing the bud when young and then splitting broadly into two or three triangular segments. *Capitula* 4–6 mm, depressed-hemispheric. Involucres subequaling flowers at anthesis, and opening broadly at maturity; involucral bracts 2–3-seriate, the outer bracts triangular-ovate, greenish to uniformly dark brown, shaggy-ciliate on margins and villous in two submedial bands. Floral trichomes obtuse to clavate, strongly tuberculate. *Flowers* ca. 20–24 per capitulum, the pistillate flowers peripheral, the staminate central, with 14–18 pistillate flowers to 6 staminate flowers (in two capitula sampled). Receptacle sparingly long-pilose with brownish hairs. Receptacular bracts subequaling flowers, linear-subspatulate, the apex slightly cucullate, pubescent as sepals, the base sharply carinate. *Pistillate flowers*: Pedicels 0.3–0.45 mm long, fine and membranous. *Sepals* broadly obovate-spatulate to subtruncate at apex, sometimes weakly cymbiform, 1.55–1.65 mm long × 0.65–0.8 mm wide at middle, 0.15–0.2 mm wide at base, blackish-brown, short-ciliate (apical cilia to 0.17 mm) along upper margin, and appressed-long-pilose in two bands flanking the upper dorsum, the basal half of the midrib hygroscopically thickened, spadiceous-brown in fruit, and recurving when dry, the broad upper half of the sepal remaining chartaceous, erect; sepals detaching from fruit on dispersal. *Petals* spatulate, obtuse, brownish at tip, 1.25–1.35 mm long, the widest in a flower ca. 0.65 mm wide, ca. 2.2 times longer than wide, bearing scale-like staminodes at base, densely long-pilose with long tuberculate hairs on abaxial upper half except for midvein, also tufted subapically within, the hairs enfolding the style branch, petals not thickening in fruit, dispersed with fruit. *Gynoecium* with style base 0.35 mm long; nectaries ca. 0.7 mm long, dark red-brown, penicillate to subclavate-infundibular, with fringe of colorless stiff-walled papillae at mouth, these rigidulous and maintaining shape after anthesis; style branches 0.85 mm long, thick and dark red-brown, non-involute. Only two slightly misshapen seeds seen, 0.6–0.63 mm long, pinkish to red-brown, the pseudotrichomes weak. *Staminate flowers*: Pedicels 0.35–0.4 mm, membranous, nearly glabrous. *Sepals* 1.6–1.7 mm × 0.5–0.6 mm, spatulate to obrhombic or subtruncate, color and pubescence as in the female flowers, narrowed toward base and shallowly fused. *Corolla* 1.8 mm long; the anthophore 1.15 mm long, comprising ca. 65% the length of the corolla, grading from 0.15 mm wide at base to 0.35 mm wide at apex; the tube 0.65 mm long including well-defined brownish-tinged lobes. Filaments brownish-tinged above; exsert not more than 0.3 mm beyond lobe tips, anthers persistent, cream to light brownish. Nectaries equaling corolla sinuses.

#### Phenology.

Collected in flower May, September, October.

#### Distribution.

Endemic to Peru, Piura, Cordillera de Huancabamba, District Carmen de la Frontera (Fig. 8).

#### Habitat.

Grass páramo (or *jalca*), probably of anthropic origin, and “burnt cloud forest, growing under *Pteridium aquilinum*” (*Weigend & Dostert 98/252*). Elevation ca. 2900–3000 m.

#### Conservation status.

Assessed as Critically Endangered, according to IUCN Criteria B1ab(iii) (IUCN 2014). Known from one locality in an unprotected area subject to deforestation, subsistence agriculture, and tourism.

#### Notes.

*Paepalanthus huancabambensis* is similar in habit and dimensions to *Paepalanthus dendroides*, but differs by its very lax, elongate peduncle sheaths well exsert from the leaf mat, and the large capitula with more flowers. It also differs in the dark blue-green leaf color, compared to the consistently pale green leaves of the widespread *Paepalanthus dendroides*, and preliminary anatomical study distinguishes it from that species by the presence of adaxial vein buttresses (bundle sheath extensions) in leaf median section. The broadly spatulate densely pilose female petals are similar to those of *Paepalanthus dendroides*. However, the longer style base, the dark rigidulous nectaries with stiff colorless papillae fringing the rim, and the size of the nectaries relative to the corolla tube in the male flowers all suggest *Paepalanthus pilosus*.

Except for the lax peduncle sheaths, this species lacks any strong distinctive features of its own but its mixture of critical characters prevent it from being easily placed in any related species, and do not immediately suggest hybrid origin. It is endemic to the Cordillera de Huancabamba near the border of Peru and Ecuador in the western part of the Andean chain. Notably, in the same vicinity are also found an atypical form of *Paepalanthus pilosus* (*Cano 16840*, discussed under Paepalanthus pilosus var. pilosus), and at higher elevations the only known populations of *Paepalanthus lodiculoides* from Peru and Ecuador.

#### Additional specimens examined.

**PERU. Piura**: Huancabamba, Cordillera Chinguela (Sapalache el Cármen), 2900 m, 15 Sept 1981, *Sagástegui et al. 10225* (HUT n.v., MO), Huancabamba-Sapalache, waterfall E of Chorro Blanco E of Sapalache, growing under *Pteridium aquilinum* in burnt cloud forest, 2650–2930 m, 19 May 1998, *Weigend & Dostert 98/252* (M, USM n.v.)

**Figure 9.**
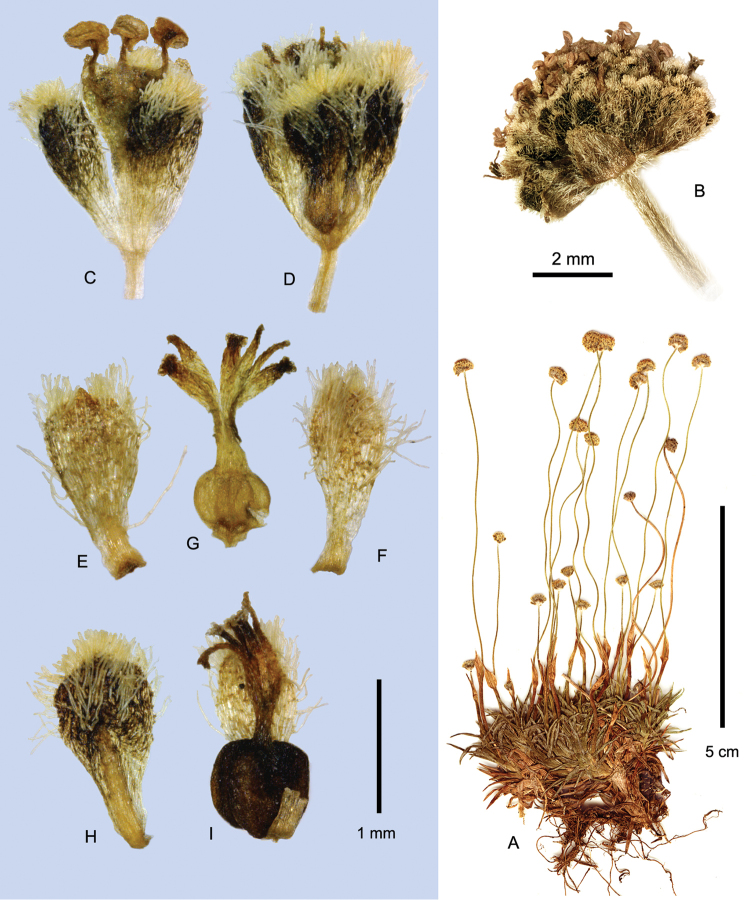
*Paepalanthus huancabambensis* (*Sagástegui 16799*). **A** Habit **B** Capitulum, anthesis **C** Staminate flower, anthesis **D−I** Pistillate flower **D** Whole flower **E** Petal, adaxial **F** Petal, abaxial **G** Gynoecium, juvenile **H** Sepal, anthesis **I** Gynoecium and one petal, young fruit.

### 4. Paepalanthus lodiculoides

Taxon classificationPlantaePoalesEriocaulaceae

Moldenke

Figs 2E , 10

Paepalanthus lodiculoides Moldenke, Bull. Torrey Bot. Club 68: 68. 1941 [31 Dec 1940].
Syngonanthus steyermarkii Moldenke, Phytologia 2: 418. 1948. Type: Venezuela. Táchira: Páramo de Tamá, near Venezuelan-Colombian border, 3045–3475 m, 15 July 1944, *J. Steyermark 57372* (holotype: NY [NY00103700]; isotype: F).
Paepalanthus polytrichoides var. densus Moldenke, Phytologia 8: 392. 1962. Type: Colombia. Cundinamarca: Paramo de Chisacá, around Laguna de Chisacá, 3650–3700 m, 29 Dec 1959, *J. Cuatrecasas & R. Jaramillo M. 25737* (holotype: LL; isotypes: COL [COL000006924], US [US00088389]).
Paepalanthus lodiculoides var. floccosus Moldenke, Phytologia 32: 47. 1975. Type: Colombia. Boyacá: Paramo de la Sarna, entre Sogamoso y Vado Hondo, 5 km al NE de la Laguna de Tota, 3510 m, 30 Mar 1973, *A. Cleef et al. 9214* (holotype: COL [COL000006910]; isotype: LL, U [U0007809], US [US00088365]).

#### Type.

Colombia. Boyacá: Nevado de Cocuy, Alto Valle de las Lagunillas, 4000 m, 12 Sept 1938, *J. Cuatrecasas & H.García Barriga 1537* (holotype: US [US00088366]; isotypes: BC [BC638454], COL [COL000006909], F, NY [NY00102897], P [P00741963]).

#### Description.

Plants compact densely branched mosslike cushions, reportedly up to 23 cm in diameter (*Soderstrom 1262*) and 2 cm high (Pedraza-Peñalosa et al. 2005). *Leaves* 1.7–5 (–6.5) mm long including broad basal sheath, the sheath often comprising half or more of the leaf length, the lamina subulate, dark to pale green, ca. 0.25–0.35 mm wide in the middle when mature, apex sharply acute (Loja) to subacute or minutely rounded; leaves densely congested along the short stems, and often half buried in deep woolly pubescence of the stem and lower leaf cilia, the tips glabrous. *Peduncles* (2–) 5–11 mm long, usually exsert 1 mm or more from sheaths and leaf mat at anthesis, obscurely 3-costate and often scurfy-pilose in lower half, rigid, subterete, and glabrous at apex. *Peduncle sheaths* (1.7–) 3–5 mm long, with an oblique scarious, sharp-acuminate to irregular or bifid apex, glabrous or obscurely tufted apically, margins eciliate (but *cf.* Syngonanthus steyermarkii type, see below). *Capitula* (1.4–) 2.5–3 mm in diameter. Involucres about equaling flowers; involucral bracts ovate to orbicular-ovate, dark black-brown throughout or paler brown along midvein, tufted at apex with clavate to linear, smooth to slightly tuberculate trichomes. *Flowers* ca. 4–6 per capitulum, sex ratio of capitula varying widely, from flowers all male to mostly female to some mixture of the two, even on same plant, the few flowers mostly peripheral, subtended by broad upper involucral bracts; receptacular bracts only rarely produced, these narrower and more oblong than involucral bracts, and carinate at base. *Pistillate flowers*: Pedicels sclerified, blackish, 0.1–0.15 mm, persisting on receptacle as bumps. *Sepals* broadly elliptic to suborbicular, strongly rounded-cymbiform in fruit, 1.2–1.7 mm long by ca. 0.65 mm wide at middle, 0.35–0.45 mm wide at base, deep blackish brown, sometimes with a pale medial streak, tufted with trichomes at apex. *Petals* oblong-obovate to broadly spatulate, acute-erose to acuminate, 1.1–1.6 mm × 0.4–0.7 mm, cream to nearly black, the distal half moderately pilose on both surfaces in two submedial or submarginal bands. *Gynoecium* at anthesis with ovary ca. 0.3 mm, style column 0.3 mm, nectaries ca. 0.35–0.6 mm, the glandular portion about equaling the stalk, clavate, the papillae soft and membranous, concentrated at apex but scattered along outside, colorless or tinged orange-brown at base, style branches 0.7–0.9 mm, brownish. Seeds 0.55–0.6 mm long, reticulate with short pseudotrichomes; locule wall thin, dehiscent or in some specimens observed adhering to the seeds, and the locules splitting apart without dehiscing (perhaps only in dry material). *Staminate flowers*: Pedicels 0.15–0.25 mm, brown. Sepals broadly spatulate, blackish toward apex, tufted; corolla with narrow membranous anthophore and broad tube with acute to acuminate lobes; anthers cream, exsert; the nectaries only half-equaling the corolla sinuses.

#### Phenology.

In Peru, flowering May–June (early in dry season); in Ecuador, September; in Colombia and Venezuela, July–Sept and Nov–March. Pedraza-Peñalosa et al. (2005) reports flowering times similar to *Paepalanthus pilosus* at Chisaca, corresponding to the “little dry season.”

#### Distribution.

Colombia (Eastern Cordillera); Bogotá D.C., Boyacá, Cundinamarca, Santander. Venezuela (Paramo de Tamá): Táchira. Ecuador: Loja. Peru: Piura. This is the first report of the species from Peru. (Fig. 8)

#### Habitat.

In paramo, at (3000–) 3300–4000 m. Cited by some authors as characteristic of very wet páramo of lake margins, bogs, and in grass paramo (Madriñán and Zapata 2001, Pedraza-Peñalosa et al. 2005). However Cleef (1981) reports Paepalanthus lodiculoides var. floccosus from the “upper zone of atmospherically dry [bunchgrass] páramo,” on the west side of the Eastern Cordillera, and the typical variety from “lower bamboo páramo” on atmospherically wet (east) slopes.

#### Conservation notes.

Known from two disjunct bands of paramo, one 420 km long in the north, and one about 100 km long in the south, under cool wet conditions.

#### Discussion.

*Paepalanthus lodiculoides* is easily distinguished from all other species in the group by the tiny moss-like leaves, delicate peduncles, and small capitula. The diaspores enclosed by persistent sepals and petals are similar to those of *Paepalanthus caryonauta*. According to Madriñán and Zapata (2001) this species forms larger and more compact cushions than *Paepalanthus pilosus* at Páramo Chingaza, Cundinamarca. Pedraza-Peñalosa et al. (2005) describe the cushions as less than 2 cm tall, or only about half as tall as those of sympatric *Paepalanthus pilosus* (“*Paepalanthus karstenii*”). The branch architecture is similar to the other species but the dense cushions are sometimes distinguished by the many soft, nearly upright stems tightly packed together, possibly on wetter sites. Other individuals have shorter, more rigid stems and shorter branchlets. There are also pronounced differences in the amount of stem pubescence and leaf coloration. A densely white-woolly form with long hairs on the stems and lower leaf margins was described as Paepalanthus lodiculoides var. floccosus, but treated as a synonym by Luteyn (1999). That synonymy is provisionally accepted here, since other characteristics are the same, and the difference in pubescence seems likely due to environmental variation. A photograph of mature ring-shaped cushions of the floccose form was published by Moldenke (1976, p. 50).

This species is also unique in the complex for the variable sex ratios of the capitula, which may be all staminate to mostly pistillate on the same plant. Capitula with exclusively pistillate flowers were not observed. Pedraza-Peñalosa et al. (2005) describe capitula with staminate flowers peripheral, but this was not observed in herbarium material.

The type of *Syngonanthus steyermarkii* Moldenke is the northernmost record of the species. It differs by the shorter peduncles (2–3 mm vs. 5–11 mm), shorter sheaths (1.7–2.2 mm vs. 3–5 mm) and capitula only about half the diameter of other specimens (1.4–1.5 mm vs 2.5–3 mm). The peduncle sheaths of this specimen are also abnormally developed, the apical lobe resembling an involucral bract in color and texture. Steyermark described the habitat as a limestone outcrop, which, if true, would be unusual for Eriocaulaceae, which almost universally occur on acidic substrates.

The three names cited in synonymy here were first cited as synonyms by Luteyn (1999) at my suggestion.

#### Additional specimens examined.

**COLOMBIA. Bogotá, D.C.**: Sumapaz, Páramo de Chisacá, 3900 m, 9–11 Nov 1958, *Barclay & Juajibioy 6113* (F,MO); 22 Mar 1959, *Barclay 7183* (MO); Parque Natural de Sumapaz, La Union – La Pedregal 3350–3720 m, 13–15 Jan 1997, *Betancur 6935* (MO); Páramo Chisacá, 3680–3700 m, 16 Sep 1961, *Cuatrecasas & Jaramillo 25987* (F); Andabobos, 3720–3760 m, 8 Jan 1969, *Cuatrecasas & Idrobo 27054* (F); Páramo Chisacá, 3596 m, 26 Sep 1966, *Soderstrom 1262* (MO); Lagunas de Chisacá, 3600 m, 15 Feb 1964, *Uribe 4672* (MO). **Boyacá**: Nevado del Cocuy, valle de Las Lagunillas, 4000–4300 m, 12 Sep 1938, *Cuatrecasas 1537* (F); Páramo del Pedrisco, km 270 Sogamoso – Pajarito, 3000 m, 27 Aug 1953, *Langenheim 3589* (F,MO); **Santander**: Paramo de Almorzadero, 3600 m, 1 Jan 1960, *Barclay 10412* (MO). **ECUADOR. Loja**: Muletrack Amaluza – Palanda, 3350–3450 m, 22 Sep 1976, *Øllgaard & Balslev 9717* (F). **PERU. Piura**: Huancabamba, El Talanco, 13 Jun 1961, *Acleto 572* (USM [photo]), 3400 m, 12 Jun 1961, *Friedberg 256* (USM [photo]); Huancabamba, Huaringas, 3957 m, May 1984, *H. Ochoa 13* (USM [photo]).

**Figure 10.**
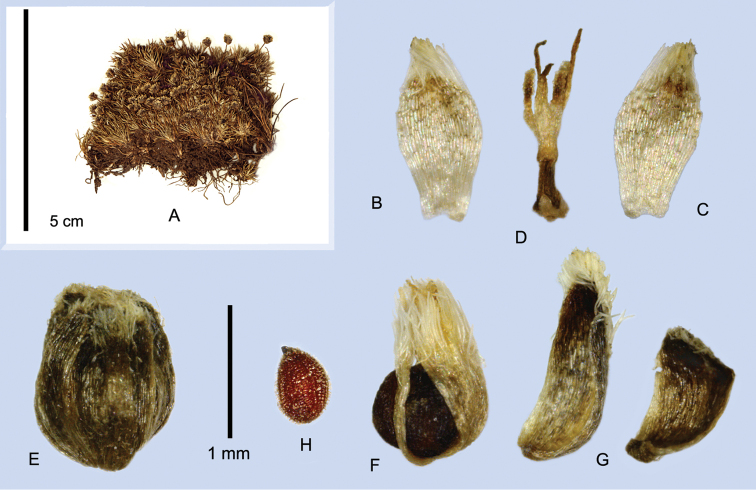
*Paepalanthus lodiculoides*. **A** Habit **B−H** Pistillate flower and fruit **B** Petal, adaxial **C** Petal, abaxial **D** Gynoecium (ovary abortive, atypical.) **E** Mature diaspore **F−G** Diaspore (**F**) with sepals removed (**G**) **H** Seed. (**A** *Cuatrecasas 25987* **B−D** *Barclay 6113* **E−G** *Øllgaard 9717* **H** *Steyermark 57372*)

### 5. Paepalanthus pilosus

Taxon classificationPlantaePoalesEriocaulaceae

(Kunth in H.B.K.) Kunth

Paepalanthus pilosus (Kunth in H.B.K.) Kunth, Enum. Pl. 3: 518. 1841.
Eriocaulon pilosum Kunth in H.B.K., Nov. Gen. Sp. (quarto ed.) 1: 251–252. 1815 [1816]. Type: Colombia. Cundinamarca: “Crescit in frigidis montanae planitiei Bogotensis inter Suba et Suacha, alt. 1340 hex.,” Jul 1801, *Bonpland & Humboldt s.n.* (lectotype, here designated: B [B_10_0243899] in part, as to plants mounted on left and right side of sheet. The plant mounted in the center of the sheet is Paepalanthus dendroides (Kunth) Kunth. Syntypes: B [B-W 2371], HAL [HAL0109743], P [P01762720]).
?Eriocaulon selaginoides Benth., Pl. Hartw. 260. 1846. Type: Colombia. Cauca: Popayán, near Laguna de Guanacas, 12,000 ft, [yr 1842], *K. T. Hartweg 1445* (holotype: K [K000640107]; isotypes: K [K000640106], B, BM [BM000793062], E [E00319822], LE [LE00001221])
?Paepalanthus selaginoides (Benth.) Körn., Fl. Bras. 3(1): 362. 1863. Type: Based on Eriocaulon selaginoides Benth.
Dupatya pilosa (Kunth) Kuntze, Revis. Gen. Pl. 2: 746. 1891. Type: Based on Eriocaulon pilosum Kunth in H.B.K.
Paepalanthus kupperi Suess., Bot. Jahrb. 72: 293, t. 3, fig. 8. 1942. Type: Costa Rica. Cartago/Limón/San José: [Cerro] Chirripó Grande, 3450 m, *W. Kupper 1315* (holotype: M [M0137218]; isotype: LL)
Paepalanthus espinosianus Moldenke, Phytologia 2: 228. 1947. Type. Ecuador Morona-Santiago: trail between Pailas and El Pan, 3400 m, 10 Sep 1943, *J.A. Steyermark 54342* (holotype: NY; isotypes: F, US [US00088348]).
Paepalanthus loxensis Moldenke, Phytologia 2: 229. 1947. Type: Ecuador. Loja: between Tambo Cachiyacu, La Entrada, and Nudo de Sabanilla, 2500–3500 m, 7 Oct 1943, *J. A. Steyermark 54452* [‘*54432*' in protologue] (holotype: NY; isotypes: F, GH [GH00028921], US [US00088367]).
Paepalanthus subsessilis Moldenke, Phytologia 2: 232.1947. Type: Venezuela. Lara: between Buenos Aires and Páramo de las Rosas, 2285–3290 m, 11 Feb 1944, *J. Steyermark 55495* (holotype: NY; isotypes: F, US [US00088407], VEN [VEN31215]).
Paepalanthus schultesii Moldenke, Bot. Mus. Leafl. 16(4): 65. 1953. **Syn. nov.** Type: Colombia. Cundinamarca: Macizo de Bogotá, Quebrada de Chapinero, 9000 ft, 24 Sep 1941, *R. E. Schultes 1024* (holotype: NY).
Paepalanthus dennisii Moldenke, Phytologia 7: 88. 1959. ‘*dennisi*.’ Type citation corrected by Moldenke, Phytologia 7: 120. 1959. **Syn. nov.** Type: Venezuela Merida: Mucubaji, Sierra de Santo Domingo, 3550 m, 26 Aug 1958, *R. W. G. Dennis s.n.* (holotype: K [K000640179]; isotype: LL).
Paepalanthus karstenii var. minimus Moldenke, Phytologia 30: 15. 1975. **Syn. nov.** Type: Colombia. Cundinamarca: Laguna de Verjón, 27 Jul 1917, *Bro. Aristé Joseph A.73* (holotype: US [US0088357]; isotype: LL).
Paepalanthus karstenii var. subsessilis (Moldenke) Moldenke, Phytologia 32: 47. 1975. Type: Based on Paepalanthus subsessilis Moldenke

#### Type.

Based on *Eriocaulon pilosum* Kunth in H.B.K.

#### Misapplied names.

*Eriocaulon microcephalum* *sensu* Standley (1937), non Kunth in H.B.K.; *Paepalanthus dendroides* sensu Madriñán and Zapata (2001), in part, *non* (Kunth in H.B.K.) Kunth; *Paepalanthus karstenii* sensu Moldenke (1952, 1975a, 1976, 1983) in large part, Espinosa (1997), Cleef (1981), Madriñán and Zapata (2001), Pedraza-Peñalosa et al. (2005), Rangel-Ch. et al. (1997), (Aubert et al. 2014a,b), among others.

### 5a. Paepalanthus pilosus (Kunth in H.B.K.) Kunth var. pilosus

Taxon classificationPlantaePoalesEriocaulaceae

**Figure 11.**
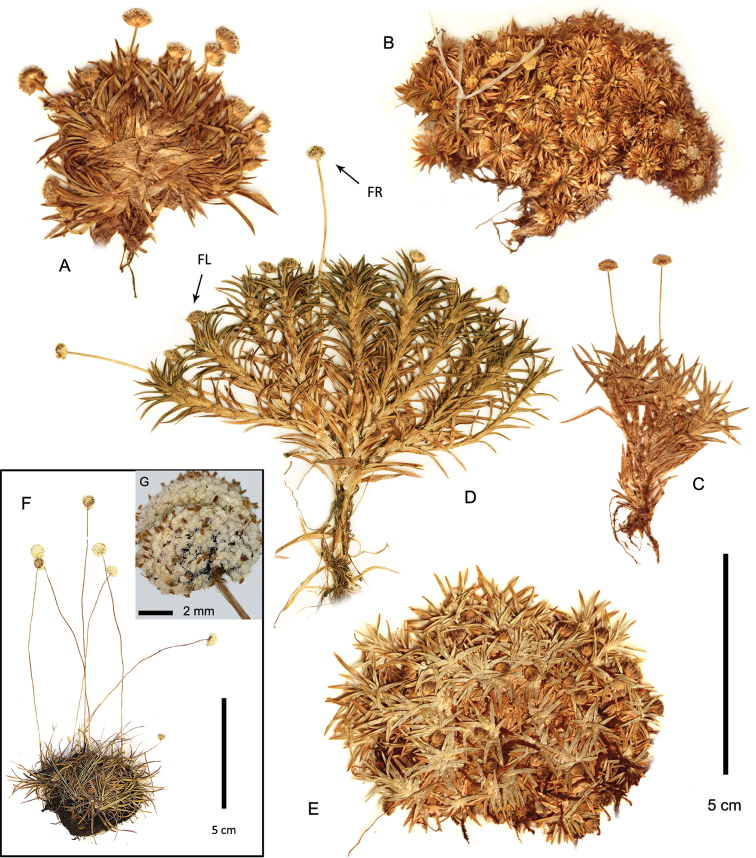
Habits of *Paepalanthus pilosus* varieties and “*Paepalanthus* sp. A.” **A−D** Paepalanthus pilosus var. pilosus. (**A** *Soderstrom 1346* **B** *Sagástegui 12242* **C** *Cano 16840* **D** *Larsen 237*.) **E** Paepalanthus pilosus var. leoniae (*León 1597)* **F−G** “*Paepalanthus* sp. A” (*Cuadros 3732*) **F** Habit **G** Capitulum. In **D** arrows indicate flowering (fl) and fruiting (fr) capitula.

**Figure 12.**
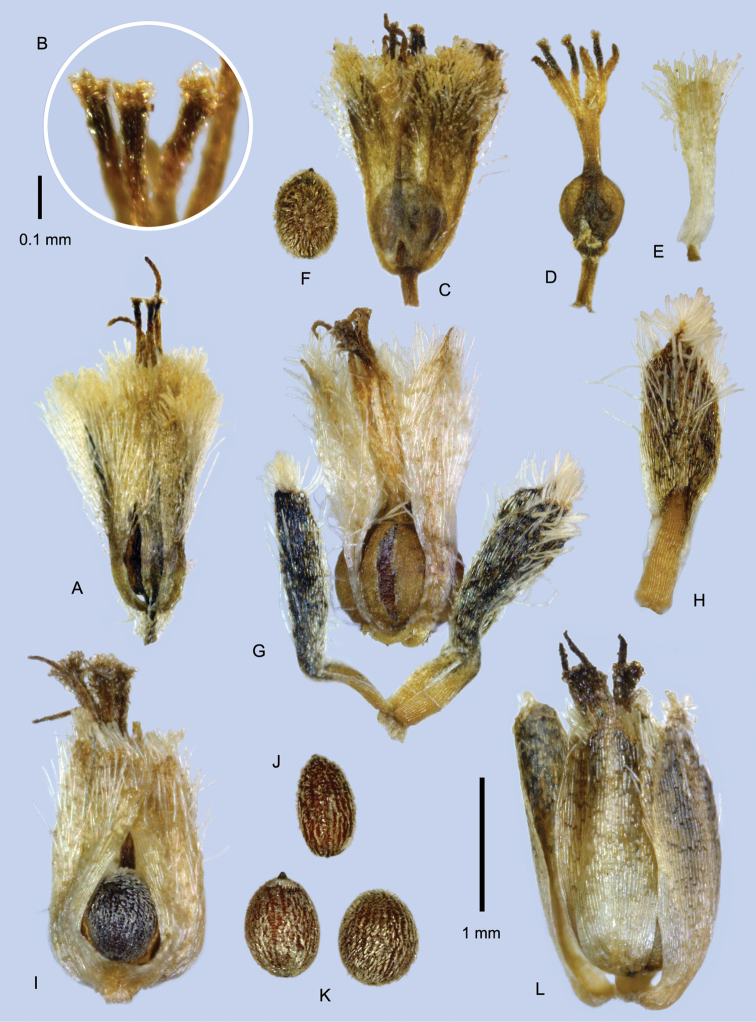
Pistillate flowers and fruits of *Paepalanthus pilosus* varieties. **A−K** Paepalanthus pilosus var. pilosus (**A−B** *Jørgensen 2366* **C−F** *Cano 16840* **G** *Cuatrecasas 25574* **H** *MacDougal 4463* **I** *Wurdack 1616* **J** *Øllgaard 9557* **K** *Boeke 2133*) **A−B** Flower in anthesis with detail of gynoecial nectaries **C** Flower in anthesis **D** Gynoecium **E** Petal **F** Seed **G** Diaspore with detached calyx **H** Sepal (fr) **I** Diaspore releasing seed **J−K** Seeds after wetting **L** Paepalanthus pilosus var. leoniae. Mature diaspore, sepals and petals intact. (*León 1597*).

Figs 1A–B ; 2D, F–J ; 3B ; 11A–D ; 12A–K

Paepalanthus pilosus (Kunth in H.B.K.) Kunth var. pilosus

#### Description.

Densely branched cushion plants, the cushions reportedly reaching 30 cm in diameter (*Molau 3223*) and up to 10 cm tall (*Berry 4366*). *Leaves* subulate, recurved, 7–16 (–20) mm long × (0.5–) 0.7–1.3 (–1.5) mm wide at midpoint; apex cuspidate-acute to short-aristate, the sharp tip evident in adaxial view; usually appressed pilose adaxially near tip (in Peru and Ecuador), the hairs smooth to roughened, in northern part of range usually with conspicuous long coarse scattered cilia to apex (rare in Peru and Ecuador), often early glabrate, bright green, texture chartaceous to rigidulous, the abaxial surface smooth or with nerves salient. *Peduncles* appressed-pilose when young especially at apex, often only 1.5–8 mm long and surpassed by the sheaths at anthesis, but frequently and variably elongating up to ten or more times this length (20–100 mm) in fruit; in Peru and Ecuador only rarely up to 50 mm at anthesis and strongly exsert (*Cano 16840*); the *sheaths* 3–15 mm long, scarious and glabrous or minutely tufted at apex, splitting apically into 2 or 3 triangular segments. *Capitula* 3–6 mm in diameter, often borne among the leaves at anthesis, frequently exsert in fruit, more rarely exsert at flowering. *Involucral bracts* equaling the capitulum to slightly surpassing it, the outer bracts 1.6–4.2 mm long, ovate to often triangular, pale gold or greenish especially along midvein, pilose on upper back to merely tufted or glabrate, the inner bracts more broadly ovate to triangular, sometimes tinged grayish-brown on shoulders; receptacle long-pilose. Trichomes of bract and sepal apices subacute to bulbous, obscurely tuberculate. Flowers ca.10–16 per capitulum, the pistillate peripheral, usually 1–2 (–3) times as many as the staminate flowers. *Pistillate flowers*: Pedicels 0.35–0.5 mm, fine or rarely wide and spongy, not thickened-rigid in fruit. *Sepals* elliptic to obovate or oblanceolate, apex acute to rounded, the base linear-ligulate to narrowly cuneate, 1.4–2.65 mm long, 0.3–1.1 mm wide at middle, 0.15–0.3 (–0.4) mm wide at base; usually tinged dusky brown above, rarely uniformly pale cream (*Sagastegui 12242*), short-tufted at apex, ciliate at upper margins, and usually pilose either side of the dorsal midvein; the basal half of the midrib hygroscopically thickened, spadiceous-brown in fruit and recurving when dry, the margins and distal half of the sepal remaining chartaceous, erect; sepals detaching from diaspore before dispersal. *Petals* oblanceolate-spatulate, acute to obtuse to emarginate, rarely (*Wurdack 1616*) broadly cuneiform; apex truncate-emarginate, apiculate; 1.5–2.35 mm long × 0.3–0.75 (–1.0) mm wide, ca. 2–6 times longer than wide, cream to brownish-tinged, pubescence similar to *Paepalanthus caryonauta*; usually not thickening in fruit, dispersed with the fruit. *Gynoecium* with style base 0.5–1.05 mm, nectaries with stalks 0.3–0.6 mm long, glandular portion dark red to brown, 0.3–0.4 mm long, penicillate with stiff whitish papillae at upper margin (northernmost Peru and northwards), or 0.4–0.5 mm long, clavate-infundibular with stiff whitish sometimes elongate papillae distributed along the outer surface, densest at the rim (Cajamarca: Celendín, Amazonas: Chachapoyas); papillae rigidulous after anthesis; styles 0.8–1.1 mm long (*Barbour 3427*: ﻿1.4–1.6 mm long), dark red-brown. Seeds 0.55–0.8 mm long, red-brown, reticulate with abundant white erect to suberect pseudotrichomes. *Staminate flowers*: Pedicels 0.2–0.5 mm. *Sepals* 1.2–2.45 mm, fused at base unequally, from very briefly to about 1/3 sepal length, obovate to usually spatulate, acute to rounded, narrowed to base and not thickening with age. *Corolla* 1.2–2.5 mm long, the anthophore 0.5–1.5 mm long, comprising 30–65 % the corolla length, membranous and 0.1–0.2 mm diameter at base, broadening to 0.2–0.3 mm near apex; the corolla tube including lobes 0.5–1.65 mm long. Filaments similar to *Paepalanthus caryonauta*, usually dark red-brown between apex and the base of the corolla lobe, exsert ca.0.2–0.5 mm beyond the lobes. Nectaries reaching the sinuses of the corolla tube.

#### Phenology.

In Central America, mostly collected January to May, and in July, coinciding with the main dry season, and possibly a shorter second dry season (*veranillo*), respectively (Grayum et al. 2004). In the climatically wet paramos of Colombia and Venezuela, peak flowering is June to December, generally the wet season (Rangel-Ch. 2000; Cuello et al. 2010; Vareschi 1970), though probably coinciding with short dry periods, as observed by Pedraza-Peñalosa et al. (2005). In Peru, flowering and fruiting mostly from July to September, during a pronounced dry season; in Ecuador, also during predominantly dry months from September to November (to January), fruiting through April.

#### Distribution.

Costa Rica (Cerro Talamanca): Cartago, Limón, San José. Panama: Bocas del Toro. Colombia (Eastern Cordillera, Sierra Nevada de Santa Marta, possibly Serrania de Perijá, also rare in Western and Central Cordilleras): Antioquia, Bogotá D.C., Boyacá, Cauca, Cundinamarca, Magdalena, Norte de Santander, Santander. Venezuela (Cordillera de Merida and Ramal de Guaramacal): Apure, Lara(?), Merida, Táchira, Trujillo. Ecuador: Azuay, Loja, Morona-Santiago, Zamora-Chinchipe(?). Peru (to 7° S lat.): Amazonas, Cajamarca, Lambayeque, Piura. (Fig. 6)

#### Habitat and ecology.

Common and characteristic ground cover (*rasante*) or cushion species of wet páramo, rarely subpáramo, on boggy organic soils, or at pond and bog margins but not in standing water (Schmidt-Mumm and Vargas-Rios 2012); often associated with *Sphagnum*, usually growing amongst shrubs or bunchgrass, in Colombia frequently also with bamboo (*Chusquea tessellata* Munro) or *Espeletia* spp. (Rangel-Ch. et al. 1997; Rangel-Ch. and Ariza 2000; Vargas and Rivera 1991). It is reported by some observers as growing at the base of bunchgrass (Franco-Rosselli et al. 1986) or shrubs (Weberbauer 1945) or under rock ledges, but is also reported in full sun. In Ecuador and the western cordillera of Peru, it is occasionally reported from montane forest. Elevation (2700–) 3000–4000 (–4400) m.

*Paepalanthus pilosus* is a commonly cited species in phytosociological analyses of North Andean paramo, usually under the name *Paepalanthus karstenii* (Rangel-Ch. et al. 1997). It is reported as a “characteristic and dominant species” of the alliance *Paepalantho karsteni* – *Chusquioni tessellatae* in wet páramo east of Bogotá (Rangel-Ch. and Ariza 2000). It is tolerant of disturbance and may even have a minor role in succession, being reported by several collectors as locally abundant on wet banks and roadsides, and in regenerating burnt paramo. Cleef (1981) noted that it is one of the first species after *Castilleja* to colonize burnt bunchgrass páramo. Cuello and Cleef (2009), in Venezuela, found it occurring abundantly in *Sphagnum* mats around the margins of an old lakebed undergoing succession to bunchgrass páramo, and suggested that this new azonal alliance (*Sphagno-recurvi* – *Paepalanthion-pilosi* Cuello & Cleef) was associated with disturbance by wildlife fragmenting the *Sphagnum* mat. Vargas and Rivera (1991, quoted by Rangel-Ch. et al. 1997) found *Paepalanthus pilosus* in paramo disturbed by grazing cavies and rabbits. One collector reported that the plant forms an abundant ‘*necromasa*’ with good water-retaining properties relevant to peat formation (*Bernal 1647*).

#### Conservation status.

The status of this widespread variable taxon, reported as a colonizer tolerant of disturbance, is presumed to be of Least Concern (IUCN 2014).

#### Taxonomic history.

No type material is found for *Eriocaulon pilosum* Kunth in the herbarium P-Bonpl. in Paris (Stauffer et al. 2012). The lectotype specimen chosen from Kunth’s herbarium (B) includes two individuals of *Paepalanthus pilosus*, with one short-pedunculate individual of *Paepalanthus dendroides* mounted between them. The middle plant is a good match for the type of *Eriocaulon dendroides* Kunth, described from the same locality. Kunth’s concepts of the two species were based partly on the shorter peduncles in *Paepalanthus pilosus*, but this character varies in both species. Kunth also differentiated *Eriocaulon pilosum* by leaves rigid, “pilose-ciliate,” with a sharp apex, and involucral bracts ovate, acute, while *Eriocaulon dendroides* was described as having leaves acuminate, membranous and glabrous, and involucral bracts obovate. These characters are adequate to distinguish the two elements on the sheet and to justify exclusion of the middle plant from the type material.

*Paepalanthus pilosus* has suffered confusing taxonomic treatment over time. Körnicke (1863) recognized *Paepalanthus pilosus* and *Paepalanthus dendroides* (both described from Bogotá) as well as *Paepalanthus selaginoides* (Popayán) as distinct taxa, distinguishing *Paepalanthus selaginoides* by the near obsolete peduncles, and *Paepalanthus pilosus* from *Paepalanthus dendroides* by the robust scattered cilia of the leaf margin. Ruhland (1903) synonymized all three under *Paepalanthus pilosus* with the claim that these diagnostic characters were too variable, sometimes even within specimens, an impression perhaps fostered by the mixed sheet of *Paepalanthus dendroides* and *Paepalanthus pilosus* from Kunth’s herbarium. At the same time, Ruhland erected an additional new species, *Paepalanthus karstenii*, also from near Bogotá, distinguished from *Paepalanthus pilosus* by the “leaf indument and apex,” the involucral bracts broad and glabrous abaxially, and “a different form of the perianth.” Inexplicably, he also described capitula as 2–3 mm wide in *Paepalanthus pilosus* versus 6–8 mm wide in *Paepalanthus karstenii*, which accords neither with the type of *Paepalanthus pilosus* (capitula 6.5 mm) nor earlier descriptions. How Ruhland thought the leaf indument, apex, or perianth in *Paepalanthus karstenii* differed from that of *Paepalanthus pilosus* is not clear from his description, leaving only the key character of bract pubescence, which also varies widely within species. In fact the broad (obovate) subglabrous bracts seen in the type of *Paepalanthus karstenii* are more typical of *Paepalanthus dendroides* as recognized by both Kunth and Körnicke. The identity of *Paepalanthus karstenii* needs further study (see Doubtful Taxa).

Moldenke (1975b) ostensibly followed Ruhland, treating *Paepalanthus dendroides* as a synonym of *Paepalanthus pilosus*, and distinguishing *Paepalanthus karstenii* by the “involucral bracts glabrous on the outer surface,” but his use of the names in annotations (ca.1930's–1980's) doesn’t correlate with bract pubescence or shape. In his pattern of annotations, Moldenke revived the appropriate distinction between *Paepalanthus dendroides* and *Paepalanthus pilosus*, but confused the nomenclature, mostly annotating typical *Paepalanthus pilosus* as *Paepalanthus karstenii*, while applying the name *Paepalanthus pilosus* to *Paepalanthus dendroides* and occasional long-pedunculate individuals of *Paepalanthus pilosus*. This convention was followed by later authors (e.g., Cleef 1981, Madriñán and Zapata 2001). Huft’s treatment (1994) was similar but treated Central American *Paepalanthus pilosus* as *Paepalanthus kupperi* rather than *Paepalanthus karstenii*. Hensold and Hammel (2003), in a treatment of Costa Rican species, re-established *Paepalanthus dendroides* as a distinct taxon and corrected application of the name *Paepalanthus pilosus* to accord with the original concept of Kunth and Körnicke, which includes both *Paepalanthus kupperi* and most material determined as *Paepalanthus karstenii*.

Moldenke did not specify distinguishing characters for his other species here placed in synonymy of *Paepalanthus pilosus*, nor were these names widely used. Of his new infraspecific taxa, Paepalanthus karstenii f. corei was said to differ from that species by the peduncles only 1–2 cm in length, and Paepalanthus karstenii var. minimus by both leaves and peduncles shorter. The type of the former is in fact *Paepalanthus dendroides*, though Moldenke most commonly applied the name to *Paepalanthus pilosus* and *Paepalanthus caryonauta*.

*Paepalanthus espinosianus* and *Paepalanthus loxensis* were first treated in synonymy of *Paepalanthus pilosus* by León-Yánez and Hensold (1999), and *Paepalanthus subsessilis* by Hensold (2008). *Paepalanthus selaginoides* was originally placed in synonymy of *Paepalanthus pilosus* by Ruhland (1903) and is clearly closest to that species, but represents the only record of the species from the Central Cordillera of Colombia. Pending closer examination of the type, its placement here is provisional.

#### Discussion.

*Paepalanthus pilosus* in Peru and southern Ecuador is characterized by the usually dwarf flowering peduncles with capitula subsessile at anthesis, and the outer involucral bracts greenish or with a green midvein and often longer than wide. In this area, soft hirsutulous pubescence of the upper leaf surface near the apex, similar to that found in *Paepalanthus dendroides*, is common. From Costa Rica to Colombia and Venezuela, long robust scattered cilia along the distal margin are frequent and diagnostic when they occur (hence the species epithet; *cf.* Fig. 2D), but rare in our area. These cilia can often be detected even in older glabrate leaves due to the persistent enlarged basal cells. From *Paepalanthus dendroides*, *Paepalanthus pilosus* may also be distinguished by the relatively narrow elliptic to oblanceolate petals, and the prominent dark rigid nectaries; and from *Paepalanthus caryonauta* by the usually larger capitula, the sharply acute to aristate leaf tips, and (in the typical variety) by the form of the fruiting calyx. (See Table 1.)

Habit varies from a dense compact mat pressed horizontally by collectors (as in the type of *Paepalanthus espinosianus* Moldenke), to a rounded cushion with branch lengths of a few centimeters, pressed and mounted vertically (as in the type of *Paepalanthus loxensis* Moldenke) The superficial difference in aspect of these two forms caused Moldenke to ally the former species with *Paepalanthus karstenii*, but the latter species with *Paepalanthus glaziovii* Ruhland (subsect. *Dichocladus*). This character almost certainly varies in response to habitat, as Heilborn (1925) noted in the vegetatively similar species *Plantago rigida* Kunth, which forms rounded cushions on wet sites and flat mats on drier sites. Leaves may be ascending to strongly reflexed and appressed to the ground, the latter perhaps a response to drier or sunnier conditions. An example are the collections *Hernandez-Schmidt 1330* and *1432* from the same locality in Cundinamarca, the former reported on rocks in full sun, with leaves reflexed close to ground, the latter on saturated soil, with leaves erect.

Peduncle length is highly variable, as already noted by Ruhland (1903), but much of this variation may be developmental. Actively flowering capitula are usually borne on peduncles only a few millimeters long, barely emergent from the sheath, and invested by the sharp cuspidate tips of subtending foliage leaves. Dramatic peduncle elongation often occurs later in fruit, as shown by specimens bearing both subsessile flowering capitula at stem tips, and fruiting capitula lower on the stem on peduncles up to ten times as long (e.g., *Larsen 237*, Fig. 11D). The delayed timing of peduncle elongation may afford wind and frost protection during anthesis, while later enhancing fruit dispersal. Peduncle elongation doesn’t always accompany fruiting or may be minimal, but even in specimens with many subsessile fruiting capitula still embedded in the leaf mat, occasional remains of much longer peduncles can be found (e.g. *Barbour 3427*, *Sagastegui 12242*). Notably, the only specimen from Peru and Ecuador with peduncles already elongate (2–5 cm) at anthesis was also the only specimen reportedly collected from “montane forest,” where it was said to be rare (*Cano 16840*). This plant may represent a taxonomically distinct variant, but for now is treated as an environmental form. In Colombian populations of the Eastern Cordillera, early peduncle elongation may be more common.

Other characters showing wide variation in Peru and Ecuador are involucral bract length, capitulum size, and flower and seed size. For example, the type of *Paepalanthus loxensis* (Loja) contrasts sharply with that of *Paepalanthus espinosianus* (Azuay) by its much smaller involucral bracts, capitula, flowers, and seeds. Indeed, in most plants from Azuay, in the Cerro Fierro-Urcu (Loja), and in some localities in Peru (Jaén, Bagua, Celendín), outer involucral bracts are ca.2.6–4.0 mm, with the tips often surpassing the large capitula 4–6 mm in diameter (*cf.* Fig. 2H). In contrast, most specimens from southern Loja, the western Cordillera of Peru, and southern Amazonas, Peru, have involucral bracts about 1.6–2.6 mm long and capitula 3–4 mm in diameter (Fig. 2I–J). The length of the pistillate flower sepals was measured in 18 specimens of Peru and Ecuador and was imperfectly correlated with capitulum size. Small flowers (sepals 1.4–1.8 mm) were found in the small-capitate plants from southern Loja and northwestern Peru, and a few large-capitate plants of Azuay; all other specimens were large-flowered (sepals ca. 1.8–2.4 mm long). Seed size showed the strongest geographic correlation, with the smallest seeds observed in the small-flowered specimens from southern Loja and the western cordillera of Peru (0.53–0.63 mm, 5 samples), while elsewhere seeds were larger (0.67–0.8 mm, 8 samples). Among the large-flowered collections, those of Amazonas, Peru, south of the mouth of the Rio Utcubamba (ca. 6° S latitude) are distinguished by the unusually broad female petals (l/w ratio 1.8–2.9 vs. 3.5–7.0 elsewhere), broadly rounded to even truncate-emarginate at apex (Fig. 12I), and by an ambiguous pattern of sepal thickening, both characters suggesting intermediacy with Paepalanthus pilosus var. leoniae, of San Martín. Also shared with that variety are the funnelform nectaries (Fig. 12I) with unusually large, spreading papillae (also found in *Sagastegui 12242*, Celendín), in comparison to the commonly penicillate nectaries with small papillae found elsewhere.

*Paepalanthus pilosus* in Colombia requires further careful study. In the Eastern Cordillera, particularly in disturbed paramo near Bogotá, there are more pronounced morphological extremes in the species than in other parts of its distribution. Here, leaf lengths range up to 3 cm, capitula from 3.5–8 mm in diameter, peduncles are occasionally strongly exsert at flowering, and involucral bract color and form are variable, ranging from nearly obovate-rounded to narrowly triangular, and pale gold to dark pigmented on the shoulders. Madriñán and Zapata (2001) indicate two sympatric elements for the Páramo Chingaza, one with ciliate leaves and peduncles exceeding the leaves in length (“*Paepalanthus dendroides*”), and one with leaves nearly glabrous and peduncles shorter (“*Paepalanthus karstenii*”). However type material of both *Paepalanthus pilosus* and *Paepalanthus karstenii* have prominent scattered cilia of the leaf margins, and both have variable peduncle lengths, which may be quite long in fruit. Judging from the photographs, both elements from Páramo Chingaza fit within my working concept of *Paepalanthus pilosus*, but the presence of two reproductively isolated crypto-taxa in this area cannot be ruled out. Some differences especially in leaf size, orientation and pubescence may be due to microhabitat, but polymorphism does seem more exaggerated in collections from the vicinity of Bogotá than from elsewhere, and collectors have often recognized two elements at one locality (e.g., *Barclay 4031*, *4057*, Paramo de Guasca; *Dwyer 8186*, *8189*, Paramo Cruz Verde; *Rangel 4045*, *4048*, Boyacá, Duitama). For simplicity I have treated as *Paepalanthus pilosus* all forms with subulate, sharply cuspidate leaves and the flower and fruit structure described above, and consider robust spreading cilia of the distal leaf margin also diagnostic when present, as it is in *Paepalanthus pilosus* of Venezuela and Central America. This may however obscure a more complicated taxonomic picture.

Hybridization may be a factor contributing to the chaotic variation in *Paepalanthus pilosus* in the vicinity of Bogotá. Some material, such as the type of *Paepalanthus karstenii* (see Doubtful Taxa) looks intermediate with *Paepalanthus dendroides*, as to elongate peduncles and rounded involucral bracts. Typical *Paepalanthus dendroides* was originally described from Bogotá, but has not been collected in the vicinity since 1917, while it is still widespread elsewhere. In addition, probable hybrids between *Paepalanthus pilosus* and *Paepalanthus caryonauta* have been collected at the margins of the range of *Paepalanthus pilosus*, at Paramo Frontino (Antioquia), and on the eastern slope of Paramo Sumapaz, south of Bogotá (see *Paepalanthus caryonauta* discussion).

A final complication in the taxonomy of Colombian *Paepalanthus pilosus* is the presence in the Serranía del Perijá and vicinity of a closely related but more robust taxon, which may intergrade with it, discussed below under *Paepalanthus* sp. A.

#### Selected specimens examined

(of 122 total). **COSTA RICA. Cartago**: Cerro Asunción, 3350 m, 19 Mar 1978, *Wilbur 26123* (F). **Limón**: Parque Nac. Chirripó, Valle Las Morrenas, 9°29'24"N, 83°29'24"W, 3500 m, *E. Alfaro 417* (MO). **San José**: Canton de Dota, Cerro Vueltas, 9°37'40"N, 83°51'10"W, 3150 m, 28 Mar 1994, *Cascante et al. 212* (F,MO); Cerro Chirripó, 9°27'36"N, 83°29'24"W, 3400–3739 m, 27 Jul 1996, *Gamboa R. et al. 490* (MO). **PANAMA. Bocas del Toro**: Cerro Fábrega, 9°07'N, 82°52'40"W, 3100–3300 m, 6–8 Mar 1984, *Davidse et al. 25303* (F,MO). **COLOMBIA. Antioquia**: Mpio. Urrao, Paramo Frontino, near Llano Grande, 3450 m, 27 Oct 1976, *Boeke & McElroy 265* (NY [01347937] p.p., mixed with *Paepalanthus caryonauta* × *Paepalanthus pilosus* at top center), *Ibidem*, ﻿3320–3450 m, 2 Mar 1989, *MacDougal & Roldán 4463* (MO). **Bogotá, DC**: Entre Alto de las Cruces-La Viga y Diego Largo, 3380–3320 m, 19 Jun 1939, *Cuatrecasas 5553* (F); Páramo de Chisacá, 3660-3720 m, 11 Sep 1961, *Cuatrecasas & Jaramillo 25882* (F); Páramo de Cruz Verde, 3300–3500 m, 20 Sep 1917, *Pennell 2074* (F,MO); Paramo de Sumapaz, 3750 m, 8 Aug 2003, *S. Rios et al. 16* (UDBC [UDBC15910]). Cerro Monserrate, 26 Apr 1951, *Romero-Castaneda 2492* (F); Páramo de Chisacá, 3596 m, 15 Oct 1966, *Soderstrom 1346* (MO); Usme, Predio El Refugio EAAB, alt. 3726 m, 2 Aug 2011, *Trujillo 600* (UDBC [UDBC30513]). **Boyacá**: Nevado del Cocuy, valle de Las Lagunillas 4000–4300 m, 12 Sep 1938, *Cuatrecasas 1501* (F); Paramo de la Rusia, Duitama – Charalá, 3624 m, 20 Aug 1953, *Langenheim 3508* (MICH); Duitama, Paramo de Belen, 06°01'35"N, 72° 57'08"W, 3900 m, 30 Sep 1986, *Rangel et al. 4045* (MO). **Cundinamarca**: Paramo de Guasca, 3000–3400 m, 13 Jun 1957, *Barclay 4031* (MO); Represa de Neusa, 3500 m, 22 Jun 1957, *Barclay 4150* (MO); Boqueron de Chipaque, 3350 m, 12 Jul 1957, *Barclay 4489* (MO); Páramo de Choachi, 3400 m, 18 Jun 1959, *Barclay 7757* (MO); Mpio. de Fómeque, Sector La Playa (Parque Nac. Natural Chingaza), 3180 m, 31 Aug 1991, *Bernal et al. 1647* (COL [COL000066835]); La Calera, Paramo de Palacio, 3420–3500 m, 8 Dec 1959, *Cuatrecasas et al. 25574* (F); Mpio. Subachoque, Paramo El Tablazo, 3400 m, 6 Oct 2003, *M. Hernandez Schmidt et al. 1330* (COL [COL000034189]), 12 Dec 2003, *M. Hernandez Schmidt 1432* (COL [COL000034188]); Sopó, 2700 m, 14 Feb 1951, *Schultes 11590* (MO). **Magdalena**: Sierra Nevada de Santa Marta, Laguna Chubdula, 10°55'N, 73°53'W, 3480 m, 29 Jul 1972, *Kirkbride & Forero 1784* (F); Slopes of Cuchilla Chebachucua, valley of Rio Duriameina, 10°38'N, 73°38'W, 3500 m, 31 May 1977, *S. White & Alverson 642* (MO). **Norte de Santander**: Cucutilla, Páramo El Romeral, 3200–3800 m, 25 Feb 2002, *Ortiz R. et al. 978* (COL [COL000066965]); **Santander**: Paramo del Almorzadero, 3600 m, 1 Jan 1960, *Barclay 10394* (MO). **VENEZUELA. Apure**: Distr. Paéz, paramo de Tamá, 3100 m, 26 Jun 1973, *Ruiz-Teran & López-Figueiras 8555* (MICH). **Lara/Trujillo**: Parque Nacional Dinira, Paramo de Jabón, vista al Tocuyo, 09°33'55"N, 70° 06'18"W, 3000 m, 13 Aug 1999, *Riina et al. 522* (F). **Merida**: Distr. Rangel, Sierra de Santo Domingo, paramo de Mucubaji, 3560–3600 m, 19 Nov 1959, *Barclay & Juajibioy 9565* (MO); Distr. Justo Briceño, Alto del Totumo, hoya del Río Chirurí, a 19.5 km de El Aguila, 3900–4000 m, 11 Oct 1983, *Berry 4229* (F); Distr. Campo Elias, San Jose de Acequias – Mucutuy, 3120 m, 22 Aug 1984, *Berry 4366* (F,MO); Distr. Rangel, Paramo de Mucuchies, 3750 m, Sept 1952, *Humbert 26300* (F); Distr. Miranda, Timotes – Paramito, 2285–3500 m, 24 Mar 1944, *Steyermark 55727* (F); Distr. Libertador, La Aguada, 3475 m, 17 Dec 1969, *Steyermark & Koyama 102359* (F,MO). **Táchira**: Paramo de Tamá, Pata de Judio, 3100–3500 m, 19 Oct 1978, *Luteyn et al. 5927* (MO). **Trujillo**: Parque Nac. Guaramacal, 2800–3100 m, 15 Jun 2001, *Dorr et al. 9005* (F). **ECUADOR. Azuay**: Paramo de Matanga, 03°13'42"S, 78°57'14"W, 3340 m, 18 Jul 2006, *Aedo et al. 13036* (MO); Gualaceo – Limón, side road at km 25.2, 03°00'S, 78° 40'W, 3540–3600 m, 12 Jan 2000, *Jørgensen et al. 1851* (F,MO); Sigsig – Gualaquiza, km 28, 03° 11'48"S, 78°47'09"W, 3280–3330 m, 14 Nov 2000, *Jørgensen et al. 2366* (F,MO). **Azuay/Morona-Santiago**: Muletrack Sevilla de Oro-Mendez (Paramo de Castillo), 2°48'S, 78°36'W, 3250–3500 m, 16 Sep 1976, *Øllgaard & Balslev 9557* (F). **Loja**: Tambo de Savanilla, 18 Oct 1876, *André K-1438* (F); Fierro Urco, Saraguro-Loja, 03°43'10"S, 79° 19'18"W, 3840 m, 6 Dec 1994, *Jørgensen et al. 1241* (MO); Yangana – Valladolid, 2800–2900 m, 5 Sep 1985, *Larsen & Dall 237* (MO), *Ibid.*, 3150 m, 14 Nov 1997, *Lewis & Klitgaard 3726* (MO). **PERU. Amazonas**: Prov. Bagua, Cordillera Colán 3290 m, 9 Sep 1978, *Barbour 3427* (F,MO); Prov. Chachapoyas, Leimebamba-Chilchos trail, 4 Jul 1977, *Boeke 2133* (MO,USM n.v.); Prov. Luya, Distrito Camporedondo, Tullanya, 06°04'29"S, 78°18'44"W, 3320 m, 8 Dec 1996, *R. Vásquez-M. & Rojas 21996* (MO); Prov. Chachapoyas, 3000 m, *Weberbauer 4416* (F frag ex G); Molinopampa – Diosan pass, 3100–3300 m, 5 Aug 1962, *Wurdack 1616* (F). **Cajamarca**: Prov. Jaén, Sallique, Localidad El Páramo, 05°40'51"S, 79°19'00"W, 3400 m, 22 Jul 1998, *Campos et al. 5334* (F,MO); Prov. Celendín, Jalca de Kumulca, 3300 m, 18 Aug 1984, *Sagástegui et al. 12242* (F,MO). **Lambayeque**: Ferreñafe, Cañaris, 3253 m, 22 Apr 2010, *Chocce et al. 5688* (USM [photo]); **Piura**: Huancabamba, Carmen de la Frontera, Nueva York, 3160 m, 28 Jul 2006, *Cano et al. 16840* (F,USM [photo]).

### 5b. Paepalanthus pilosus var. leoniae

Taxon classificationPlantaePoalesEriocaulaceae

Hensold var. nov.

urn:lsid:ipni.org:names:77155459-1

Figs 11E , 12L

#### Diagnosis.

Differs from the typical variety by the capitula 2–3 mm in diameter, flowers 5-10 per capitulum, the pistillate flowers with sepals elliptic, uniformly thickened in fruit and dispersed with the fruit; petals of pistillate flowers oblong to oblong-spatulate with broadly rounded to truncate apex.

#### Type.

PERU. San Martín: Prov. Mariscal Caceres, NW sector Rio Abiseo National Park, grassland in Paredones, [07°40'16.73"S, 77°29'1.78"W], 3600 m, 16 March 1988, *B. León & K. Young 1597* (Holotype: MO!).

#### Description.

*Leaves* 5.5–12 mm long × 0.6–1.4 mm wide at midpoint, sharply cuspidate, finely appressed-ciliate near apex when young, early glabrate. Peduncles 3–6.5 mm long, fruiting peduncles the same length in specimens observed, the sheaths 5–7 mm. *Capitula* 2–3 mm in diameter, the involucres obconic or cupulate in flower and fruit, not opening broadly, the bracts similar to typical variety. Flowers 5–10 per capitulum, the staminate equal in number to pistillate or in fewer-flowered capitula the proportion of staminate flowers may be reduced. *Pistillate flowers*: Pedicels 0.25–0.35 mm. *Sepals* elliptic, obtuse to rounded, 1.35–2.4 mm long × 0.65–0.8 mm wide at middle, 0.25–0.35 mm wide at base, pale brownish, tinged dusky-brown on shoulders, becoming uniformly thickened and cymbiform at maturity, enclosing the fruit and dispersed with it. *Petals* oblong to oblong-spatulate, with apex broadly rounded to truncate, retuse or apiculate, 1.5–2.0 mm long × 0.65–0.85 mm wide, 2.2–2.5 times longer than wide, pilose as in the typical variety or with trichomes restricted to subapical tufts, uniformly thickening in fruit, as the sepals. *Gynoecium* with style base 0.5–0.65 mm long, nectaries 0.65–1.0 mm long, papillae at upper margin colorless, stiff, globose (*Leon 1597*) to linear (*Young 4368*); style branches 0.75–1.7 mm. Seeds not seen but mature ovary locules 0.65 mm (*Young 4368*), 0.75 mm (*Leon 1597*). *Staminate flowers*: Pedicels 0.25–0.35 mm. Sepals 1.35–2.1 mm long, fused 35–55 % of their length. Corolla 1.55–2.05 mm long, with the anthophore 0.85–1.15 mm long, comprising 45–75% the length of the whole corolla, ca. 0.1–0.25 mm in diameter at base, the tube and lobes 0.4–1.05 mm long. Filaments brownish toward apex; nectaries reaching corolla sinuses.

#### Etymology.

Named in honor of Dr. Blanca León, of the Universidad Nacional Mayor de San Marcos and the University of Texas, a generous and accomplished botanist whose many contributions include ecological and taxonomic study of Rio Abiseo National Park.

#### Phenology.

Collected in March and July. A mild dry season occurs June to August (Young and Leon 1988).

#### Distribution.

Endemic to Peru, San Martín, Prov. Mariscal Caceres, Rio Abiseo National Park. (Fig. 6)

#### Habitat.

From high-elevation grasslands, at 3450–3800 m. Young and León (1988) compare the habitat to the wet páramos of southern Ecuador, and note that the U-shaped valleys at high elevations where this variety was collected were glaciated as recently as 12,000 years ago.

#### Conservation status.

Endangered, Criteria B1ab(iii) (IUCN 2014). This variety is known from only two sites 10 km apart in the protected area of Rio Abiseo National Park In recent years, cattle-grazing, which may have been a threat, has ceased (B. León, pers. comm.) However, the very small distribution, and the fact that this variety represents the southernmost occurrence of a wetland páramo species suggests vulnerability to climate fluctuations.

#### Discussion.

This variety is found at the southernmost and highest elevation station for the species in Peru. It occurs above 3400 m at the very south end of the Amotape-Huancabamba floristic zone as characterized by Weigend (2004), in the drainage of the Rio Huallaga. This compares to the typical variety collected at up to 3400 m, in the drainage of the Rio Marañón.

The uniformly thickened fruiting perianth of this variety is similar to that of *Paepalanthus caryonauta*, complicating the otherwise tidy distinction between the two species. However the size and shape of the leaves, the dwarf peduncles, the pale greenish involucres, and the large rigid nectary papillae, all suggest *Paepalanthus pilosus*, and the type specimen in particular looks nearly identical to populations of typical *Paepalanthus pilosus* from adjacent southern Amazonas. In fact, these populations are partly intermediate in floral morphology between the two varieties, as discussed under the typical variety. Given the variation in *Paepalanthus pilosus* in Peru and the contiguity of distribution, it seems best for now to treat this taxon as a variety of that species.

The specimen *Young & León 4368*, collected ca. 10 km from the other two, is marked by the very small size (5.5–7 mm) of the dark green leaves. The flowers and seeds are also smaller. The habitats of the two localities differed, with the type locality a typical patchy wet páramo among outcrops, while the small-leaved plant was in a broad boggy area bordered by small trees, where cattle once pastured (B. León, pers. comm.)

#### Additional specimens examined.

Perú. San Martín: Prov. Mariscal Caceres. Puerta del Monte, northwest corner of Rio Abiseo National Park; high elevation grassland on bottom of u-shaped valley; [7°39'30.20"S; 77°28'13.67"W], 3450 m, 10 Jul 1987, *K. Young & B. León 4368* (F); Pastizales de Empedrada, entre manojos de Calamagrostis, [07°40'17"S, 77°29'2" W], 3750–3785 m, 27 Jul 2000, *B. León & K. Young 4579* (USM [photo!]).

### Paepalanthus Species A

Taxon classificationPlantaePoalesEriocaulaceae

Figs 6 , 11F–G

#### Misapplied (?) name.

*Paepalanthus macarenensis* *sensu* Moldenke (1983) in part, and Rivera-Díaz (2010), probably *non* Moldenke (1952).

This robust taxon has a clumping habit structurally similar to *Paepalanthus pilosus* but lacks the dense pulviniform aspect. Its other similarities include acute to aristate leaves, scarious splitting peduncle sheath tips, pale gold lanceolate involucral bracts, and a nearly identical flower and fruit morphology. It differs by the following characters:

Leaves longer, narrowly linear-lanceolate, 3–4 cm long, the peduncles 10–20 cm long at anthesis, and the capitula 6–7.5 mm wide, much more floriferous (> 40 flowers) than *Paepalanthus pilosus* and sometimes globose at maturity, with alternating whorls of staminate and pistillate flowers. In addition, the capitula are “indeterminate,” with floral primordia found at the center of capitulum at the time of anthesis of the outer whorls. This contrasts with *Paepalanthus pilosus*, in which pistillate flowers are limited to the outer whorl, staminate to the inner, and no floral primordia are found at the start of anthesis.

The most robust individuals are found in the northern part of Serrania del Perija (ca. 10°15'– 10°20' N), but similar smaller plants are also found at the north end of the main Cordillera Oriental, about 300 km to the south. These have leaf, peduncle and sheath lengths approaching those of the Perijá plants, and in spite of their small capitula, the flowers are more numerous than in typical *Paepalanthus pilosus* (up to 40 per capitulum), and pistillate and staminate whorls alternate in the capitulum. It isn’t clear whether to treat these plants as small individuals of *Paepalanthus* sp. A, or intermediates with *Paepalanthus pilosus*. Smaller individuals are also found at Sa. de Perijá (*Cuatrecasas 25027*, *25143*, US), but were only observed from scans.

The plants of Perijá had been distributed in part as *Paepalanthus macarenensis*, a species otherwise only known from ca. 800 m in the Sierra de la Macarena (Meta). I have seen an image of the *Paepalanthus macarenensis* type, and do not believe it is the same species or closely related, but pending closer examination treat the Perijá plants only provisionally here.

#### Specimens examined.

**COLOMBIA. Cesar**: [Serranía de Perijá], Paramo de Sabana Rubia, 3250 m, 22 Jul 1987, *H. Cuadros 3732* (MO), east of Manaure, Sabana Rubia, paramo, 3000–3100 m, 6–8 Nov 1959, *J. Cuatrecasas & R. Romero-Castaneda 25025* (COL [COL000223802], US n.v.), La Paz, Corr. San Jose de Oriente, Vda. Altos del Riecito – Altos de Perijá, Fca. Los Sauces, 10°14'48.75"N 72°57'44.5"W, 3096 m, 27 Feb 2006, *J. O. Rangel 13692* (COL [COL000254069])

#### Smaller individuals.

**COLOMBIA. Norte de Santander**: de La Laguna a Nariz de Judío (Mutiscua), 19 Jun 1946, *M. de Garganta 1209* (F); **Santander**: Paramo de Las Vegas, 3700–3800 m, 20–21 Dec 1926, *E. P. Killip & A. C. Smith 15626* (F); Paramo de Santurban, between Tona and Mutiscua, 3800–4300 m, 18 Feb 1927, *E. P. Killip & A. C. Smith 19557* (F).

### Doubtful taxon

#### Paepalanthus karstenii

Taxon classificationPlantaePoalesEriocaulaceae

Ruhl.

Paepalanthus karstenii Ruhl., Pflanzenr. IV. 30: 155. 1903. Type: Colombia. Cundinamarca: Páramo de Chipaque, [yrs 1848–1856], *H. Karsten s.n.* (holotype: W, destroyed [F neg. no. 29991]; lectotype, here designated: B [B_10_0243950]; isotype: LL [LL00374731]).
Dupatya karstenii (Ruhl.) Gleason, Bull. Torrey Bot. Club 53: 195. 1925.

##### Type.

Based on *Paepalanthus karstenii* Ruhl.

These unusual specimens with large capitula, broadly rounded bracts, coarsely ciliate leaves, and irregular leaf lengths appear intermediate between *Paepalanthus pilosus* and *Paepalanthus dendroides* as to bract characters and habit, and were collected at an elevation where distributions would potentially overlap. A topotype (Paramo de Chipaque, 3000 m, Dec 1855, *J. J. Triana 1022-5*, COL [COL 000302503]), det. *Paepalanthus dendroides* by Killip, has similar features but even wider variability. This may be a variant of *Paepalanthus pilosus* or a hybrid taxon and needs closer study.

### Invalid names

*Eriocaulon caulescens* Willd. mscr., non Poir. nec Hook.f. & Thomson ex Thwaites. Based on: Humboldt & Bonpland s.n. (B-Willd. 2366 [BW02366000]), annotated as *Eriocaulon caulescens* on sheet and folder. Presumed syntype of *Eriocaulon dendroides* Kunth in H.B.K.

= ***Paepalanthus dendroides*** (Kunth) Kunth

*Eriocaulon parvum* Ruíz & Pav. nom. nud., non Körn. Cited by H. Ruíz (1940, p. 230). Annotated sheets: PERU. [Huánuco:] Saxiapata, *Ruíz & Pavón s.n.* (MA [MA810392, MA810393, MA810394], probable duplicate, MO, acc. no.1612102). Collected “in the montana of Pillao and Chacahuasi,” Aug–Sept 1787, according to Ruíz (1940).

= ***Paepalanthus dendroides*** (Kunth) Kunth

### Excluded taxon

Paepalanthus pilosus var. microcephalus Moldenke, Phytologia 55(6): 372. 1984.

= ***Paepalanthus lamarckii*** Kunth, in Paepalanthus ser Leptocephali (Ruhl.) Giul.

Synonymy according to Huft (1994).

## Supplementary Material

XML Treatment for Paepalanthus subsect. Cryptanthella

XML Treatment for Paepalanthus caryonauta

XML Treatment for Paepalanthus dendroides

XML Treatment for Paepalanthus huancabambensis

XML Treatment for Paepalanthus lodiculoides

XML Treatment for Paepalanthus pilosus

XML Treatment for Paepalanthus pilosus (Kunth in H.B.K.) Kunth var. pilosus

XML Treatment for Paepalanthus pilosus var. leoniae

XML Treatment for Paepalanthus Species A

XML Treatment for Paepalanthus karstenii

## Supplementary materials

Supplementary material 1
*Paepalanthus pilosus* complex, exsiccatae list Data type: Text file This dataset is made available under the Open Database License (http://opendatacommons.org/licenses/odbl/1.0/). The Open Database License (ODbL) is a license agreement intended to allow users to freely share, modify, and use this Dataset while maintaining this same freedom for others, provided that the original source and author(s) are credited.Nancy Hensold

Supplementary material 2
*Paepalanthus pilosus* complex, specimen database Data type: Excel spreadsheet. List of examined specimens and geo-references of the localities Explanation note: Contains complete label information of all examined specimens, as well as estimated geographic coordinates (in decimal degrees) for all specimens. This dataset is made available under the Open Database License (http://opendatacommons.org/licenses/odbl/1.0/). The Open Database License (ODbL) is a license agreement intended to allow users to freely share, modify, and use this Dataset while maintaining this same freedom for others, provided that the original source and author(s) are credited.Nancy Hensold
