# Supplementary material for: The Andean Paepalanthus pilosus complex (Eriocaulaceae): a revision with three new taxa
Source: PhytoKeys. 2016 Jun 13;(64):1–57. doi: 10.3897/phytokeys.64.6864 (PMC4956916; doi:10.3897/phytokeys.64.6864)
Supplement: Supplementary material 1 — Paepalanthus pilosus complex, exsiccatae list [file phytokeys-064-001-s001.docx]

**Index to Numbered Collections**

(Notes: First collector listed only. Citations of “pilosus” refer to P. pilosus var. pilosus. Type specimens of accepted names or synonyms indicated with *.)

Acleto, C. 572 (lodiculoides).

Aedo, C. 13036 (pilosus).

Alfaro, E. 417, 1633 (pilosus).

Alston, A. H. G. 7333 (dendroides).

André, É.-F. K-1438 (pilosus).

Antonio, T. 1571 (dendroides).

Aristé Joseph, Bro. A.73* (pilosus)

Aristeguieta, L. 2442 (pilosus).

Baca, A. Y-118 (caryonauta).

Barbour, P. 3427 (pilosus).

Barclay, H. G. 4031, 4057, 4093, 4150, 4489 (pilosus); 5136, 5176 (caryonauta); 5730, 5847 (dendroides); 6097 (pilosus); 6113, 7183 (lodiculoides); 7432 (pilosus); 7629 (dendroides); 7757, 8656 (pilosus); 9374 (caryonauta); 9565, 10394 (pilosus); 10412 (lodiculoides).

Barkley, F. A. 18A100* (dendroides).

Bernal, H. Y. 1647 (pilosus).

Berry, P. 4229, 4346, 4366, 4392 (pilosus).

Betancur, J. C. 150 (pilosus); 5854 (dendroides); 6935 (lodiculoides)

Boeke, J. D. 265 p.p. (caryonauta × pilosus); 265 p.p. (pilosus); 269 (caryonauta × pilosus), 803 (caryonauta); 2133 (pilosus).

Boyle, B. 4219 (caryonauta).

Callejas, R. 10839, 11608 (dendroides).

Campos, J. 5334 (pilosus).

Cano, A. 3361 (dendroides); 3872, 3873a (caryonauta); 4024 (caryonauta); 4437, 4465 p.p. (dendroides); 4465 p.p. (caryonauta); 5152 (dendroides); 16840 (pilosus).

Cascante, A. 212 (pilosus).

Chocce, M. 5688 (pilosus).

Cleef, A. 9214* (lodiculoides).

Core, E. L. 272A* (dendroides).

Crow, G. 7400 (pilosus)

Cuadros, H. 3732 (“Species A”).

Cuatrecasas, J. 401, 1209, 1501 (pilosus); 1537* (lodiculoides); 1559A, 1612, 5553, 9502, 9980 (pilosus); 10302 (caryonauta aff. or hybrid); 10455 (pilosus); 11866 (dendroides); 19099, 20278 (caryonauta); 23654 (dendroides); 25025 (species A); 25027, 25143 (cf. species A / pilosus); 25574 (pilosus); 25737* (lodiculoides); 25882 (pilosus); 25987, 27054 (lodiculoides).

Davidse, G. 25303, 25419 (pilosus); 25871, 25928, 28991, 29339 (dendroides).

Diaz-Piedrahita,S. 2608 (cf. caryonauta × pilosus).

Dorr,L. J. 5185, 5317, 9005 (pilosus).

Dudley, T. R. 11060, 11194 (caryonauta).

Dwyer, J. D. 8186, 8189 (pilosus).

Espinal, L. S. 3310 (dendroides).

Farney, C. 901 (dendroides).

Fassett, N. C. 25629, 25633, 25929 (dendroides).

Fonnegra, R. 4650, 5408 (dendroides).

Friedberg, C. 256 (lodiculoides).

Fuentes, A. F. 12018, 13574 (caryonauta).

Galindo-T., R. RGT-1303 (dendroides).

Gamboa R., B. 490 (pilosus).

Garcia-Barriga, H. 8113, 16158 (pilosus).

Garganta, M. 1209 (species A aff.).

Gillett, J. M. 16589 (pilosus).

Godfrey, R. K. 66733 (pilosus).

Gómez, L. D. 22436, 22439 (dendroides); 22440 (pilosus); 22591 (dendroides).

Grant, J. 01-4041 (pilosus).

Hanselmann, D. P. 24 (pilosus).

Hartweg, K. T. 1445* (pilosus).

Heithaus, R. 245 (pilosus)

Hernandez Schmidt,M. 1330, 1432 (pilosus).

Holm, R. W 460 (pilosus)

Huft, M. J. 2163 (pilosus).

Humbert, H. 26300, 26894 (pilosus).

Humbles, J. E. 6086 (caryonauta).

Jiménez, A. 855, 2231, 3396 (pilosus).

Jørgensen, P. M. 1241, 1817, 1851, 2209, 2366 (pilosus).

Killip, E. P. 15626 (species A aff.); 16064 (dendroides); 19557 (species A aff.); 34090 (pilosus).

Kirkbride, J. H. 363, 1784 (pilosus).

Kupper, W. 1315* (pilosus)

Langenheim, J. H. 3508 (pilosus); 3589 (lodiculoides); 3688 (pilosus).

Larsen, B. B. 237 (pilosus).

Lechler, W. (ed. Hohenacker) 2206 (dendroides).

León, B. 1597* (pilosus var. leoniae); 2243 (dendroides); 2245, 2431 (caryonauta); 2683 (dendroides); 4579 (pilosus var. leoniae); s.n., USM111244 (dendroides).

Lewis, G. P. 3726 (pilosus).

Liesner, R. 8065 (pilosus).

Luteyn, J. 5927, 7755 (pilosus); 10737 (dendroides).

Macbride, J. F. 5182 (dendroides).

MacDougal, J. M. 4463 (pilosus).

Molau, U. 3223 (pilosus).

Monro, A. K. 5369 (dendroides)

Monteagudo Mendoza, A. 7938 (caryonauta × dendroides); 16143 (caryonauta).

Mori, S. 220 (pilosus)

Mutis, J. C. 2551 (pilosus).

Núñez, P. 7773 p.p. (dendroides); 7773 p.p. (caryonauta).

Ochoa T., H. 13 (lodiculoides).

Øllgaard, B. 8460 (caryonauta); 9557 (pilosus); 9717 (lodiculoides).

Ortiz R., N.Y. NYO-978 (pilosus).

Pennell, F. W. 1997 (dendroides); 2074 (pilosus); 2256 (cf. pilosus, atypical); 13864 (caryonauta); 13866 (dendroides).

Pérez-Arbeláez, E. 66 (dendroides).

Peyton, B. 914 (caryonauta).

Pinzón S., C. E. CP701104 (pilosus or pilosus × dendroides?).

Posada S., S. 18A100* (dendroides)

Rangel, J. O. 4045, 4048 (pilosus); 13692 (species A).

Riina, R. 522 (pilosus).

Ríos, S. 16 (pilosus).

Roldán, F. J. 252 (dendroides); 283 (pilosus); 402 (caryonauta × pilosus); 438 (dendroides).

Romero-Castañeda, R. 2492 (pilosus).

Ruiz & Pavón s.n., MO1612102 (dendroides).

Ruiz-Terán, L. E. 771, 6274, 6694, 8555 (pilosus).

Sagástegui, A. 10225 (huancabambensis); 12242 (pilosus); 16799* (huancabambensis).

Santa Cruz, L. 653, 2018 (dendroides).

Sarmiento, F. 128 (pilosus).

Schultes,R.E. 81, 1024*, 11590 (pilosus).

Smith, S. G. 1059 (pilosus).

Soderstrom, T. 1262 (lodiculoides); 1346 (pilosus).

Standley, P. 43637, 43830 (pilosus).

Stergios, B. 20801 (pilosus).

Steyermark, J. 54342*, 54452*, 55495*, 55727 (pilosus); 57372* (lodiculoides); 57377, 102359 (pilosus).

Stuessy, T. 5612 (dendroides).

Tate, G. H. 382 (caryonauta)

Taylor, R. J. 11738 (pilosus)

Triana, J. J. 1022-5 (see “P. karstenii”)

Trujillo, L. 600, 601 (pilosus).

Tupayachi, A. 50 (dendroides).

Uribe, L. 4672 (lodiculoides).

Valderrama, L. E. 18A164 (dendroides).

Valenzuela, L. 8117* (caryonauta).

van der Hammen, Th. 379 (pilosus).

van der Werff, H. 9162 (pilosus).

Vargas, G. 1715, 1749 (pilosus)

Vásquez-M.,R. 21996 (pilosus); 29038 (dendroides).

Weberbauer,A. 4416 (pilosus).

Weigend, M. 98/252 (huancabambensis).

Weston, A. S. 1545, 5986 (pilosus); 6164, 10151 (dendroides).

White, S. 642 (pilosus).

Wilbur, R. L. 26123 (pilosus).

Williams, L. O. 24472 (pilosus).

Williams, R. S. 842 (caryonauta).

Woytkowski, F. 567 (dendroides).

Wurdack, J. J. 1616 (pilosus).

Young, K. 4368 (pilosus var. leoniae).

Zarucchi, J. 6332 (dendroides).
